# Australian Cool-Season Pulse Seed-Borne Virus Research: 2. Bean Yellow Mosaic Virus

**DOI:** 10.3390/v17050668

**Published:** 2025-05-03

**Authors:** Roger A. C. Jones

**Affiliations:** UWA Institute of Agriculture, University of Western Australia, Crawley, WA 6009, Australia; roger.jones@uwa.edu.au

**Keywords:** Australia, history, virus diseases, seed-borne viruses, cool-season pulses, bean yellow mosaic virus, losses, epidemiology, management, main research achievements, future research priorities

## Abstract

Here, research on seed-borne virus diseases of cool-season pulses caused by bean yellow mosaic virus (BYMV) in Australia’s grain cropping regions since the 1940s is reviewed. A historical approach is taken towards all past studies involving the main cool-season pulse crops grown, lupin, faba bean, field pea, lentil and chickpea, and the minor ones, narbon bean, vetches and *Lathyrus* species. The main emphasis adopted is on describing what these studies revealed concerning BYMV biology, epidemiology and management. The field and glasshouse experimentation that enabled the development of effective phytosanitary, cultural and host resistance control strategies, supported by many image illustrations from past investigations, is emphasized. This review commences by providing brief background information and describing past studies on BYMV symptom and sequence variants, and alternative BYMV hosts. Next, as the lupin/BYMV pathosystem has been investigated in much greater depth than any other cool season pulse/BYMV pathosystem combination in Australia, what past studies using it have found is covered considerable detail under a series of nine different sub-headings. Finally, what is known about the less thoroughly investigated cool-season pulse/BYMV pathosystems, especially those involving faba bean, field pea and lentil, is reviewed under seven different sub-headings. Recommendations are provided concerning future research priorities.

## 1. Introduction

This is the second of three historical reviews of research on major seed-borne virus diseases of the cool-season pulse crops grown on the Australian continent. The main crops they affect are narrow-leafed (*Lupinus angustifolius*) and white (*L. albus*) lupin, faba bean (*Vicia faba*), field pea (*Pisum sativum*), lentil (*Lens culinaris*) and chickpea (*Cicer arietinum*). The principal minor cool-season pulse crops also affected include yellow (*L. luteus*) and pearl (*L. mutabilis*) lupin, common vetch (*V. sativa*), narbon bean (*V. narbonensis*), grass pea (*Lathyrus sativus*) and dwarf chickling (*Lathyrus cicera*) [1,2,3,4,5]. Volume 1 of the historical review series provided background information about Australia’s large cool -season pulse industry, general plant virus epidemiology and management principles and future threats to achieving successful virus disease control [5]. However, its main focus was on describing the biology, epidemiology and management of the seed-borne virus diseases caused by cucumber mosaic virus (CMV; genus *Cucumovirus*, family *Bromoviridae*) and alfalfa mosaic virus (AMV; genus *Alfamovirus*, family *Bromoviridae*), placing major emphasis on the field and glasshouse experimentation which enabled effective phytosanitary, cultural and host resistance control strategies to be devised against them. In addition, it described past Australian studies with five less important seed-borne viruses sometimes found infecting cool-season pulse crops in Australia (broad bean stain virus (genus *Comovirus*, family *Secoviridae*), broad bean true mosaic virus (genus *Comovirus*, family *Secoviridae*), broad bean wilt virus (genus *Fabavirus*, family *Secoviridae*), cowpea mild mottle virus (genus *Carlavirus*, family *Betaflexiviridae*) and peanut mottle virus (genus *Potyvirus*, family *Potyviridae*)) [5]. Here, Volume 2 provides a historical account from the 1940s up to the present time of the extensive volume of Australian research on the sometimes seriously damaging diseases resulting in major economic losses that bean yellow mosaic virus (BYMV; genus *Potyvirus*, family *Potyviridae*) causes in these cool-season pulses and how to manage them. Its main focus is on BYMV-pulse pathosystem biology, epidemiology and management, placing special emphasis on describing field and glasshouse experiments which resulted in the development of effective phytosanitary, cultural and host resistance control strategies. An extensive collection of color images of BYMV field-based research from the last 40 years is included within a series of figures to help the reader comprehend the scope of the research described and what it revealed. This volume also makes recommendations for Australia regarding what future BYMV cool season-pulse research is required. In the future, Volume 3 will focus on Australian research on the diseases that pea seed-borne mosaic virus (PSbMV; genus *Potyvirus*, family *Potyviridae*) causes.

## 2. Background Information

**Figure 1:**
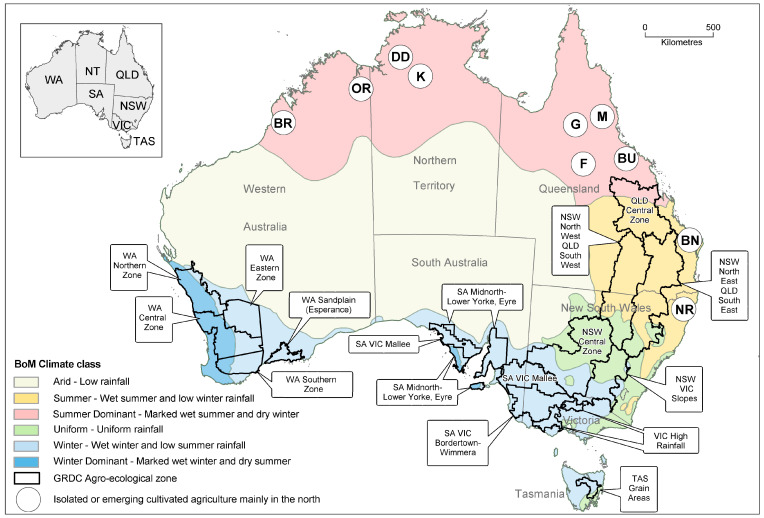
Map showing both major and minor grain producing regions where pulses are grown in Australia. The acronyms of Australian states and territories used here are WA (Western Australia), SA (South Australia), VIC (Victoria), TAS (Tasmania), NSW (New South Wales), QLD (Queensland) and NT (Northern Territory). Australian Bureau of Meteorology (BOM) climate groupings are used to distinguish the main regions where grain crops (including pulses) grow, but letters distinguish the smaller irrigated grain production regions in WA—BR (Broome) and OR—Ord River Irrigation Area, the NT—DD—Douglas/Daly and K—Katherine, QLD—BU (Burdekin), BN—Bundaberg, G—Gilbert, F—Flinders and M—Mareeba (including Atherton and Ravenshoe) and NSW—NR—Northern Rivers. Black lines delineate boundaries of distinct agro-ecological zones. Image credit@Department of Primary Industries and Regional Development/P. Goulding.

BYMV was first described in 1924 and, like CMV, was one of the earliest plant viruses studied [6,7]. When it first arrived in Australia is unknown, but the first Australian research publications that mentioned it under the synonyms *Phaseolus* virus 2 or pea mosaic virus (PMV) were in 1943, by which time it was established in two states, Victoria (VIC) and Western Australia (WA) [8,9]. It has a broad host range consisting of dicotyledonous and monocotyledonous species of flowering plants, and causes serious diseases in many legume species [1,2,10,11,12,13]. It is non-persistently transmitted by more than 20 aphid species [1,2], amongst which the legume colonizing species *Myzus persicae*, *Macrosiphum euphorbiae*, *Aphis craccivora*, *A. gossypii*, *Aulacorthum solani*, *Acyrthosiphon kondoi* and *Acyrthosiphon pisum* and the non-colonizing species *Rhopalosiphum padi* are important in Australia [2,3,14,15,16,17,18,19,20,21]. BYMV occurs in all Australian States and Territories. Cool-season and tropical pulses and pasture legumes are among the many economically significant cultivated plants that it infects. As stated in the Introduction, its cool-season pulse hosts include faba bean, field pea, lentil, narrow-leafed lupin, white lupin, yellow lupin, pearl lupin, chickpea, common vetch, narbon bean, grass pea and dwarf chickling. They also include sandplain lupin (*L. cosentinii*), bitter vetch (*V. ervilia*) and *Lathyrus clymenum* [2,3,4,22,23,24,25,26]. The regions of Australia where pulse crops are grown are shown in Figure 1. The main pulse crops in the southern grainbelt (south and central south New South Wales (NSW), South Australia (SA), Tasmania (TAS), VIC and WA) are field pea, faba bean, lupin, chickpea and lentil, whereas in the northern grainbelt (central and southern Queensland (QLD) and north and central–north NSW) they are chickpea, lentil, field pea and lupin [5]. However, differences occur within and between regions, e.g., lupin is a much bigger crop in WA than in other regions, whereas NSW only grows this pulse in its central–north region, central QLD does not grow lupin, field pea or faba bean, and chickpea is not grown in TAS [5]. Chickpea is also grown under irrigation in tropical northern Australia, and the minor cool-season pulse common vetch is grown in all climatic zones [5].

## 3. BYMV Symptom and Sequence Variants

**Figure 2:**
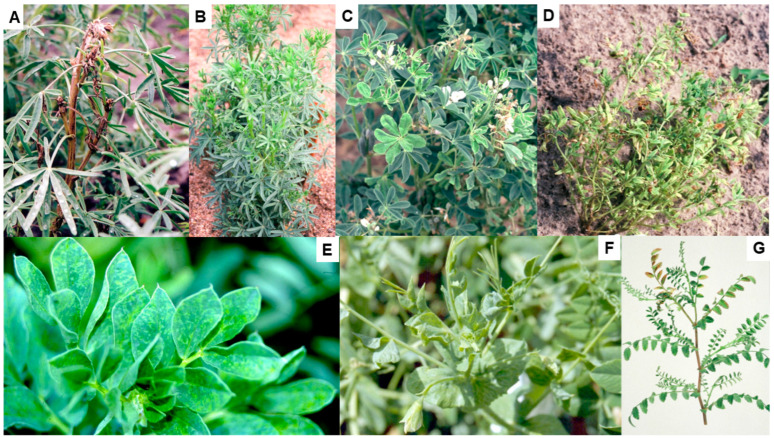
Images of disease symptoms caused by infection with bean yellow mosaic virus (BYMV) in plants of the most widely grown cool-season pulse species for Australia. (**A**) Young narrow-leafed lupin (*Lupinus angustifolius*) plant showing early-phase systemic necrotic shoot symptoms typical of infection with its necrotic strain (prior to killing the plant) (at The Lakes in 1993). (**B**) Narrow-leafed lupin plant showing upper leaf symptoms typical of infection with its non-necrotic strain consisting of mosaic, accompanied by leaflet downcurling and size reduction (at Avondale in 1999). (**C**) White lupin (*L. albus*) cv. Lutop plant with typical leaf symptoms caused by BYMV infection consisting of mosaic, deformation and size reduction (at South Perth in 1990). (**D**) Lentil (*Lens culinaris*) cv. Digger plant with typical leaf symptoms caused by BYMV infection consisting of leaf mosaic, leaflet chlorosis, downcurling and reduction in size and plant dwarfing (at South Perth in 1996). (**E**) Apical portion of faba bean (*V. faba*) plant showing typical leaf mottle symptoms caused by BYMV infection (at South Perth in 1996). (**F**) Apical portion of field pea plant (*Pisum sativum*) showing typical leaf symptoms of mosaic and leaflet deformation caused by BYMV infection (at Medina in 1988). (**G**) Apical portion of chickpea (*Cicer arietinum*) cv. Heera shoot showing leaflet reddening symptoms caused by BYMV infection (at South Perth in 1998).

The foliage symptoms that BYMV causes in infected cool-season pulse species growing in Australia vary widely, but typically involve leaf mosaic, chlorosis, deformation and size reduction, systemic necrosis and plant stunting (Figure 2A–G) (see also Table 1 in Jones and Congdon [5]). Symptoms also vary widely in other pulse hosts and in pasture legume hosts growing in Australia [1,2,3,4]. Within a BYMV-infected cool-season pulse species, foliage symptom variation can result from alterations in environmental conditions, which BYMV strain is present, the growing stage at which a plant becomes infected and the genotype (i.e., cultivar, breeding line and accession) involved. This symptom variation is discussed in more detail below in Section 4, Section 5.1, Section 5.2, Section 5.4, Section 6 and Section 6.4.

Over five decades (1945–1995), a major focus of Australian research in the states of NSW, SA and VIC was directed towards establishing the relationship between diverse BYMV isolates and variants, Phaseolus virus 2 and PMV. These studies involved comparing their symptomatology in pulse and pasture legume hosts, non-legume indicator hosts, host ranges, stabilities in infective sap, serological properties, particle sizes, cytopathology and coat protein (CP) amino acid compositions [27,28,29,30,31,32,33,34]. The overall conclusion was that all three should be considered BYMV variants. Two other SA studies then compared the nucleotide (nt) sequences of different BYMV isolates and variants obtained from diverse naturally infected host species (field pea, lupin, common bean, pasture legumes, Canna and Gladiolus) growing in collection sites widely dispersed around Australia (in NSW, VIC, SA, WA, QLD and TAS) [35,36]. Some differences in isolate host range and serology coincided with differences in nt homology, but none of these isolates and variants were considered to have nt homologies distinct enough to belong to a different virus. Nevertheless, a decade later, the issue of whether PMV was too different to be considered as a BYMV strain was revisited. When a VIC study published in 1992 compared the amino acid sequence of the CP gene of BYMV strain S (BYMV-S) with that of clover yellow vein virus (CYVV; genus *Potyvirus*, family *Potyviridae*) strain B (CYVV-B), the two were sufficiently distant to be consider distinct viruses [37]. However, in the same study, when amplified DNA probes of the 3′ non-coding regions of BYMV-S, CYVV-B and PMV ‘strain’ I (PMV-I) were used, the hybridization between each virus was weak, suggesting that all three (including PMV) were distinct viruses. Soon afterwards, another VIC study published in 1994 compared partial nt sequences of the CP gene of PMV-I with those of four BYMV strains (CS, D, S and GDD) [38]. The outcome based on nt sequence homology was that PMV-I and BYMV-CS formed a distinct BYMV sub-group and the other three BYMV strains (D, S, GDD) formed another. Thus, this study confirmed that PMV is insufficiently distinct to be considered a different virus.

Subsequent WA studies used the sequencing of BYMV’s RNA to examine the genetic diversity of its isolates from different hosts and regions of Australia. The initial focus was on comparing their CP and genome-linked viral protein (VPg) genes [39,40]. Wylie et al. [39] compared the partial CP nt sequences of 25 Australian isolates (17 new sequences) with 39 from other continents and the VPg nt sequences from eight Australian isolates (four new sequences) with six from other continents. The previous sequences came from GenBank; those from Australia were from earlier BYMV studies in WA [41,42,43] or in the Australian Capital Territory (ACT), VIC or SA [34,37,44]. Their CP phylogenetic trees had seven distinct minor phylogroups. The largest of these included dicotyledons and monocotyledons belonging to both wild and domesticated original host species. Apparently, this phylogroup was not only ‘generalist’ in terms of its natural host range, but also ‘ancestral’ to the other six phylogroups. The isolates within the other phylogroups, which all had smaller intragroup nt sequence diversity, came from domesticated plants. Two had predominantly or entirely monocotyledonous natural hosts, but the other four had entirely dicotyledonous original hosts, which included the cool-season pulses lupin, pea or faba bean. When the small number of VPg nt sequences were analyzed, there were only five phylogroups, three polytypic and two monotypic. Although their branch topologies differed from those obtained with CPs and they had greater intragroup nt diversity, there again tended to be correspondence between phylogroups and isolate original host species [39]. Later, when eight BYMV isolates from narrow-leafed lupin plants with black pod syndrome (BPS) growing in WA were sequenced, the partial CP nt sequences obtained all fitted within the main ‘generalist’ minor phylogroup [45].

Kehoe et al. [46] subsequently obtained new genomes from WA isolates of BYMV (23 isolates) and CYVV (1 isolate). The BYMV isolates sequenced were from narrow-leafed lupin plants with BPS (11) or systemic necrosis symptoms (6) and sandplain lupin plants (2) from their previous work [45] or frozen-dried specimens from narrow-leafed lupin (1), as well as *L. pilosus* (1) and faba bean (two sequences from one isolate) from another previous work [47]. The single CYVV isolate was from a frozen-dried specimen of white clover from further previous work [48]. When the complete coding regions of the 23 new Australian BYMV genomes were compared with 17 other BYMV genomes from GenBank, a phylogenetic analysis revealed nine well-supported minor BYMV phylogroups, given the names I-IX. The single CYVV isolate was grouped with a CYVV sequence from GenBank. The sequences within BYMV phylogroups I-IV were I, nine from two species each of monocots and dicots; II, seven from one monocot and two dicot species; III, three all from one monocot species; and IV, one from a dicot species, two from a monocot species and three from an unknown host species. Groups V–IX each contained three sequences solely from dicot species within the same family. With respect to original isolate sequence hosts, there was sometimes a lack of coincidence between the CP-based phylogroups of Wylie et al. [39] and those based on complete genomes [46].

Wylie and Jones [40] analyzed the complete genomic nt sequences of seven BYMV and one CYVV isolates from GenBank. They used six different recombination programs within Recombination Detection Program No. 3 (RDP3). Based on their detection by at least four out of six of these programs, they obtained evidence of eight recombination patterns. In addition, when 64 (BYMV) and 1 (CYVV) CP sequences were analyzed similarly, three recombination patterns were found. The majority of these recombinants were isolate sequences that belonged within BYMV’s large ‘generalist’ and ‘ancestral’ phylogroup, indicating that recombination might have been an important component of its evolution towards host adaptation to domesticated plants, including lupin, pea and faba bean. Subsequently, when the complete coding regions of 33 BYMV and 2 CYVV genomes were subjected to recombination analysis by RDP4, Kehoe et al. [49] found 12 firm recombination events based on detection by at least four out of seven programs. These were all in phylogroups I-VI, none being found within VII-XI or CYVV. Most of these recombination events were in sequences within phylogroups I, II, IV and VI. Interestingly, I, II and IV coincided with the former ‘ancestral’ and ‘generalist’ phylogroups of Wylie et al. [39]. Only one event was present in sequences within phylogroups III and IV. The main conclusion drawn from these results was that, in contrast to the tentative earlier deduction of Wylie and Jones [40], recombination actually favors the broadening of BYMV’s natural host range (i.e., being ‘generalist’). This was because it was widespread within sequences that comprised the three phylogroups with both monocots and dicots as natural hosts (I, II and IV). Moreover, since formerly ‘specialist’ minor phylogroups III, V and VI also contained recombinant sequences, this could enable them to ‘re-generalize’ (i.e., widen their natural host ranges). Therefore, the past association of ‘specialist’ group isolates with cultivated pulse plants in their crop global domestication centers (crops: faba bean, pea and lupin) found by Wylie et al. [39] might be in the process of being reversed by their distribution to new world regions due to increasing international trade. Such redistribution would result in mixed infections with BYMV isolates from different specialist groups leading to recombination, which in turn would enable them to ‘re-generalize’ by widening their natural host ranges.

In the future, a phylogenetic analysis of diverse Australian and other BYMV sequences is needed to provide deeper insights into its overall evolution within the continent and additional information concerning the arrival and origins of past incursions. Also, since no research has been undertaken in Australia to identify which sections of the BYMV genome encode proteins that might play roles in altering symptom expression, host adaptation or vector transmission in cool-season pulse species, this too would be a worthwhile future undertaking.

## 4. Alternative Hosts

**Figure 3:**
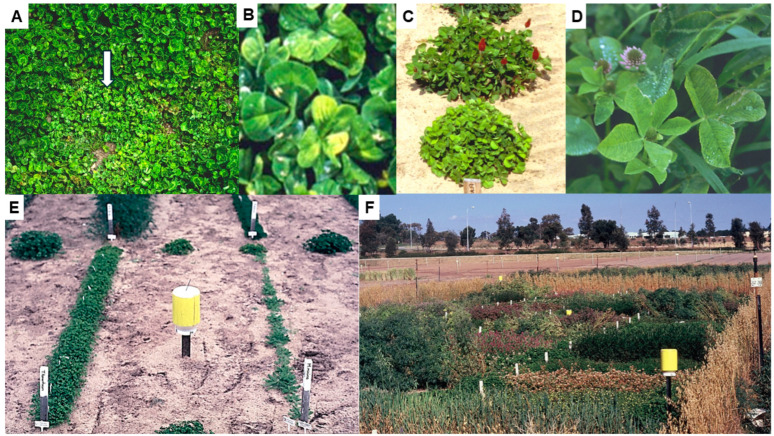
Images of disease symptoms caused by infection with bean yellow mosaic virus (BYMV) in species of alternative pasture hosts and screening procedure used to evaluate alternative host species for their susceptibilities and sensitivities to BYMV infection. (**A**) Subterranean clover (*Trifolium subterraneum*) cv. Karridale pasture with patch of stunted BYMV-infected plants indicated by white arrow (at Manjmup in 1990). (**B**) Close up of plants from stunted BYMV-infected subterranean cover patch in A showing leaflet symptoms of mottle, palor and deformation. (**C**) Individual plants within single-row plot of crimson clover cv. Caprera (*T. incarnatum*); BYMV-infected plant showing chlorosis and stunting (front) and normal-looking healthy plant (behind) (at Medina in 1996). (**D**) Plant of Moroccan clover (*T. isthmocarpum*) with obvious leaf-vein-clearing symptoms in young leaves caused by BYMV infection at (at Narikup in 1998). (**E**) Early phase of potential alternative host genotype susceptibility/resistance screening process showing stunted BYMV-infected subterranean clover transplants placed at both ends of each row to provide a uniform BYMV infection source for naturally occurring aphid vectors to spread the virus to the test rows. After they became BYMV infected, originally healthy clover transplants placed in between the infector transplants boosted the virus inoculum source for BYMV spread to the test rows. Sticky yellow traps used to monitor aphid flights (at South Perth in 1990). (**F**) Single-row plots of potential alternative host genotypes (mainly pasture legume species) surrounded by a non-host oat (*Avena sativa*) barrier undergoing BYMV susceptibility/resistance screening (at South Perth in 1990).

BYMV occurs commonly in subterranean clover (*Trifolium subterraneum*) pastures throughout Australia (Figure 3A,B) [6,7,8,9,16,22,28,50,51,52] and aphids often spread it from these pastures to pulse crops [3,53]. A list of early (mid 1950s–mid 1980s) records of BYMV infecting crop, pasture and weed host species from ACT, QLD, NSW, VIC, SA, TAS and WA was provided by Buchen-Osmond et al. [2]. In addition to subterranean clover, the non-food crop legume BYMV host species records included the annual pasture or perennial forage legumes—*Calopogonium mucunoides*, *Cassia* spp., *Centrosema pubescens*, *Crotalaria goreensis*, *Desmodium leiocarpum*, *Lotononis* sp., *Medicago murex*, *M. polymorpha*, *M. sativa* and *Trifolium semipilosum*; the Australian native legume—*Clianthus formosus*; and the ornamentals—*Gladiolus hortulanus* and *Lathyrus adoratus*. In 1989–1992, when naturalized and volunteer pasture species were sampled at sites in WA, BYMV was detected in the naturalized clover *T. cernuum* and the pasture clovers *T. balansae* and *T. vesiculosum* [51]. In 1994–1998 in WA, BYMV infection was found frequently in surveys of seed increase, evaluation and breeding plots of a range of annual pasture clover species growing at diverse locations. These clover species included the commercial pasture species *T. vesiculosum*, *T. incarnatum* (Figure 3C), *T. balansae*, *T. fragiferum*, *T. pallidum*, *T. alexandrinum*, *T. spumosum*, *T. isthmocarpum* (Figure 3D), *T. resupinatum*, *T. glanduliferum* and *T. purpureum*. They also included the naturalized pasture weed species *T. arvense*, *T. clypeatum*, *T. glomeratum*, *T. cherleri*, *T. cernuum* and *T. nigrescens* [52,54,55]. Natural BYMV hosts in WA also included the perennial native legumes *Hovea elliptica*, *H. pungens*, *Kennedia prostrata* and *K. coccinea*, *Drosera* sp. (*Droseraceae*), *Cassia* sp. (*Caesalpiniaceae*), *Triglochlin* sp. (*Hemerocallidaceae*) and the native orchids *Caladenia paludosa*, *Diuris maculata*, *Diuris longifolia*, *Microtis* sp., *Pterostylis curta* and *Thelymitra* sp. [39,40,42,51,56].

In 1989–1990 in WA, two field experiments exposed single-row plots of potential legume hosts to infection with BYMV isolate MI, which originally came from an infected *Melilotus indicus* plant [24]. In 1989, nine genotypes were replicated twice, but eight were un-replicated, whereas in 1990, two replicates of each of the thirty genotypes were tested (Figure 3E,F). All test rows were exposed to a uniform BYMV inoculum consisting of subterranean clover infector plants transplanted at both ends of each row and naturally occurring aphid vectors spread the virus to the test rows (Figure 3E). In aphid traps associated with these experiments, the main aphid vector species caught were *Myzus persicae*, *Acrythosiphon kondoi* and *Rhopalosiphum padi (*Figure 3E,F). Apart from the single genotypes tested from perennial clovers *T. repens* and *T. fragiferum*, all genotypes of every species exposed became BYMV-infected. Apart from subterranean clover, the annual pasture legumes that became infected were *Medicago aculeata*, *M. murex*, *M. polymorpha*, *M. truncatula*, *Ornithopus compressus*, *Trifolium balansae*, *T. hirtum*, *T. hybridum*, *T. pratense*, *T. resupinatum* and *T. vesiculosum*. The naturalized annual pasture clover weeds that became infected were *T. angustifolium*, *T. arvense*, *T. campestre*, *T. cernuum*, *T. clusii*, *T. dubium*, *T. glomeratum*, *T. ligusticum*, *T. scabrum*, *T. suffocatum* and *T. tomentosum*. Other legumes with BYMV infection were two common weeds *Melilotus. indica* and *M. alba*, and the pasture lupin plant *L. cosentinii*. Each genotype was allocated a relative susceptibility/resistance score based on the extent of BYMV infection in test rows. Amongst the 10 alternative host species most often present in subterranean clover pastures in WA, their rankings were ‘highly susceptible’ or ‘susceptible’ for *L. cosentini*, *T. campestre* and *T. dubium*, but ‘highly resistant’, ‘resistant’ or ‘moderately resistant’ for *T. arvense*, *T. campestre*, *T. cernuum*, *T. glomeratum*, *T. tomentosum*, *T. balansae* and *T. resupinatum* which therefore seem less likely to contribute towards BYMV spread into pulse crops. Seeds harvested from all BYMV-infected test rows were sown in the glasshouse, and their seedlings tested for BYMV seed transmission. Evidence of this occurring was only found in six species: the naturalized clovers *T. arvense* (0.1%), *T. campestre* (0.2%) and *T. glomeratum* (0.1%); the pasture species *M. polymorpha* (0.9%) and *M. truncatula* (0.3%); and the weed species *Melilotus indica* (0.5–1%) [24]. In separate glasshouse studies in WA, the transmission of BYMV isolate MI from seeds harvested from plants infected by sap inoculation to their seedlings was studied in 15 different genotypes belonging to seven annual medic species [57]. It was detected at low levels in one genotype each of *M. aculeata* (0.3%), *M. polymorpha* (1%) and *M. murex* (0.8%), but not in any of the other five genotypes belonging to these species or the eight genotypes tested of *M. rugosa*, *M. scutellata*, *M. tornata* or *M. truncatula*. These findings show that BYMV has the potential to carry over in their dormant seeds between growing seasons within annual pastures so the germination of infected seedlings can act as a primary BYMV source for the spread from annual pastures to newly planted pulse crops in the following growing season.

In 1994–1998 in WA, three field experiments studied the susceptibilities and sensitivities of alternative host species to infection with BYMV isolate MI [25]. The experimental design was that used by McKirdy and Jones [24], but there were always two replicates of each genotype present. A diverse range of pasture legumes likely to occur in close proximity to pulse crops were included; 7/18 genotypes belonging to 17 of these species received highly susceptible rankings (*Biserrula pelecinus*, *T. cherleri*, *T. clypeatum*, *T. dasyurum*, *T. incarnatum*, *T. spumosum* and *T. vesiculosum*) and the remainder were ranked as susceptible (seven: *T. glanduliferum*, *T. isthmocarpum*, *T. resupinatum*, *T. squarrosum* and *Trigonella balansae*), moderately resistant (two: *T. purpureum* and *T. michelianum*), resistant (one: *Ornithopus sativus*) or highly resistant (one: *Hedysarum coronarium*). The highly resistant genotype of *H. coronarium* proved to have extreme resistance following glasshouse inoculations with three BYMV isolates. The genotype of *Ornithopus sativus* that ranked as BYMV-resistant also ranked as BYMV-sensitive. The other 12 pasture species tested, all of which were annual pasture plants or introduced clover weed species, are potential alternative hosts from which BYMV can spread to pulse crops in the field. Amongst these, *Trifoium cherleri*, *T. incarnatum*, *T. spumosum* and *Biserrula pelecinus* were the most BYMV-susceptible and -sensitive, whereas, although ranked as susceptible, the sensitivity rankings of *T. clypeatum*, *T. dasyurum*, *T. glanduliferum*, *T. resupinatum* and *Trigonella balansae* were intermediate. Therefore, because high sensitivity to infection is associated with poor fitness leading to out-competition by a less sensitive host or non-host species in mixed-species pastures [58], less sensitive but susceptible host species seem likely to contribute most to the within-pasture virus incidence increase, and to BYMV’s subsequent spread to pulse crops. *T. subterraneum* was ranked as susceptible and highly sensitive. BYMV was seed-borne at low levels (0.03–1%) in nine of the pasture species (all pasture or naturalized clovers). These species were the pasture clovers *T. spumosum* (1%) and *T. purpureum* (0.07%); *T. vesiculosum* (0.05%), *T. michelianum* (0.05%), *T. resupinatum* (0.03%) and *T. isthmocarpum* (0.1%); and the naturalized clovers *T. clypeatum* (1%), *T. squarrosum* (0.8%) and *T. cherleri* (0.2%) [25]. Although no BYMV seed transmission was found in tests on *T. subterraneum* (subterranean clover) seed from the field experiments of McKirdy and Jones [24] and McKirdy et al. [25], it was detected occurring at 1.1% (cv. Junee) and 0.1% (cv. Trikkala) infection levels during routine tests for seed-borne virus infection on samples from pasture seed stocks undertaken in 1997 and 2006, respectively. Examples of other pasture seed stocks found infected in these two years include *T. vesiculosum* (0.05–1%), *T. incarnatum* (0.1–0.3%), *T. purpureum* (0.4%), *T. resupinatum* (0.1%), *T. squarrosum* (0.2%), *Biserulla pelecinus* (0.8%) and *Trigonella balansae* (1.2–1.4%) (R.A.C. Jones and S.J McKirdy, unpublished data). As indicated in the previous paragraph, these findings reveal how BYMV has the potential to carry over in their dormant seeds between growing seasons so infected seedlings can act as primary BYMV sources for spread from annual clover pastures to newly planted pulse crops in the next growing season.

In summary, in Australia, there are many alternative host species from which BYMV can spread to nearby cool-season pulse crops. Moreover, it can persist between annual growing seasons in the seeds of some of them, being spread by aphid vectors to nearby plants once infected seedlings germinate. These alternative host species include sown pasture legumes and naturalized pasture legumes that grow in annual pastures (especially within subterranean clover- and annual medic-based pastures), perennial forage legumes, native perennial legumes and orchids, native species belonging to other families and ornamental plants. Highly susceptible or susceptible alternative hosts are likely to be more important BYMV sources than hosts possessing BYMV resistance. However, when such hosts growing within species mixtures are also virus-sensitive rather than virus-tolerant, they are likely to be outcompeted by non-hosts. When this occurs, the virus spread diminishes so the pasture, or wild plant vegetation, becomes a less potent source for BYMV spread to nearby pulse crops. Clearly, phytosanitary measures involving the removal of BYMV-infected external and internal alternative BYMV hosts prior to sowing pulse crops constitute significant components of integrated disease management (IDM) tactics against this virus. In addition, it is important not to inadvertently sow seed of pasture legumes carrying BYMV.

## 5. Lupins

**Figure 4:**
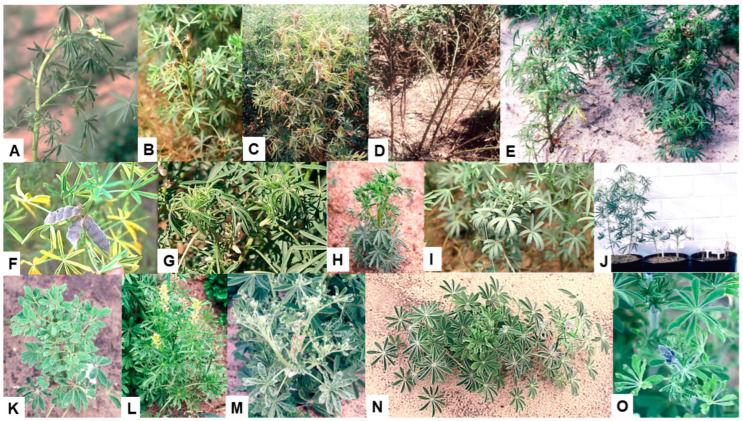
Images of disease symptoms caused by infection with bean yellow mosaic virus (BYMV) in different crop and potential crop lupin species. (**A**–**E**) Different phases of symptom development elicited by early infection of narrow-leafed lupin (*Lupinus angustifolius*) with BYMV necrotic strain. (**A**) Initial systemic ‘Shepard’s crook’ symptom consisting of bending over of the shoot tip caused by recent infection (at South Perth in 1986). (**B**) Next phase of early systemic symptom consisting of apical shoot necrosis (at The Lakes in 1993). (**C**) Later phase of infection spread consisting of apical systemic necrosis having spread to all shoots (at Woogenellup in1988). (**D**) Final phase of infection spread resulting in death of all shoots (at South Perth in 1992). (**E**) Situation where both the necrotic BYMV strain (left) and cucumber mosaic virus (right) were spreading within the same young narrow-leafed lupin cv. Gungurru stand; plant on left shows early phase of systemic necrosis symptoms whereas youngest leaves of plants on right show symptoms of leaflet chlorosis, downcurling and size reduction (plants at back healthy) (at Wongan Hills in 1995). (**F**) Late-infection BYMV necrotic strain occurring after flowering eliciting ‘black pod syndrome’ (at South Perth in 1995). (**G**–**I**) Different symptoms elicited by infection of narrow-leafed lupin with BYMV non-necrotic strain. (**G**) Early systemic symptom in upper leaves consisting of leaflet chlorosis, downcurling and size reduction (at Avondale in 1999). (**H**) Later phase of symptom development where upper portion of infected plant shows symptoms of leaf mosaic, chlorosis, deformation and size reduction, whereas lower down the leaf, appearance remains unaffected (at Avondale in 1999). (**I**) Later phase of infection occasionally observed where the only symptoms visible were upper fleshy, expanded and downcurled leaves and plant dwarfing (at The Lakes in 1993). (**J**) Glasshouse aphid transmission test with narrow-leafed lupin cv. Danja showing 2 plants killed by inoculation with necrotic strain isolate MI (right), with stunted growth caused by inoculation with non-necrotic isolate La-NN (middle) and healthy controls (left) (at South Perth in 1994). (**K**) Plant of white lupin (*L. albus*) cv. Ultra showing BYMV foliage symptoms consisting of severe leaf mottle and deformation and plant dwarfing (at South Perth in 1990). (**L**) Plant of yellow lupin (*L. luteus*) cv. Reda showing foliage symptoms of leaflet chlorosis, narrowing and reduced size (front) contrasting with larger darker green healthy leaves of neighboring plant (behind on left side) (at South Perth in 1990). (**M**) Plant of the rough-seeded lupin species, sandplain lupin (*L. cosentinii*). cv. Eragulla, showing foliage symptoms of severe leaf mottle, deformation and size reduction and plant dwarfing (front) contrasting with normal foliage of a healthy plant (behind) (at South Perth in 1989). (**N**) Plant of the rough seeded lupin species *L. pilosus* showing foliage symptoms of severe leaf mottle, deformation and size reduction in its upper leaves (center) contrasting with the foliage of heathy plants (on both sides) (at South Perth in 1987). (**O**) Plant of the rough-seeded lupin species *L. digitatus* showing foliage symptoms of severe leaf mottle, deformation and size reduction in its upper leaves and stunting (center) contrasting with the foliage of heathy plant (behind) (at South Perth in 1993).

The first report of BYMV infecting lupin in Australia was in 1939 in WA in the cultivated species narrow-leafed lupin, white lupin, yellow lupin and pearl lupin and the rough-seeded lupin species sandplain lupin and *L. pilosus* [9,22]. By 1988, it had been found infecting some of these lupin species in other Australian states: the cultivated species narrow-leafed lupin in TAS, yellow lupin in NSW and white lupin in QLD and VIC, the additional rough-seeded lupin species *L. atlanticus* and *L. digitatus* in WA and the ornamental species *L. polyphyllus* in TAS [2,3,36]. In narrow-leafed lupin, BYMV symptoms develop differently depending upon whether infection occurs early or late and the necrotic strain or the less common non-necrotic strain is present. Early infection by the necrotic strain causes the commonest phenotype, consisting of bending over of the shoot tip (‘shepherd’s crook’ symptom), necrotic stem streaking and generalized necrosis followed by plant death (Figure 2A and Figure 4A–E) [3,9,22,47,59]. However, instead of killing the plant, late infection of mature plants by this strain is limited to one or some branches that then develop BPS and/or systemic necrosis (Figure 4F) [45,60,61,62]. In contrast, necrosis is lacking when the non-necrotic strain is present as this causes a non-necrotic phenotype consisting of stunted BYMV-infected plants with leaf chlorotic mottle and leaflet downcurling or, occasionally, dead growing points with fleshy expanded leaves (Figure 2B and Figure 4G–J) [47,59,63]. Foliage symptoms in the other lupin species cultivated in Australia include, for white lupin, severe mosaic, necrotic spotting and deformation of leaves, accompanied by severe plant stunting and occasional plant death (Figure 2C and Figure 4K); for yellow lupin, narrowing of leaflets, vein clearing, mild mosaic and leaf pallor, bunchy growth and plant dwarfing (Figure 4L); for sandplain lupin and other rough-seeded lupin species, symptoms resembling those for white lupin (Figure 4M–O); and for pearl lupin, leaf vein-clearing mosaic, chlorosis and rugosity/deformation and slight plant stunting [3].

### 5.1. Necrotic and Non-Necrotic Strains

In narrow-leafed lupin plants, early BYMV infection normally causes the systemic necrosis followed by plant death phenotype described in the previous paragraph (Figure 2A and Figure 4A–E) [3,9,22]. However, commencing in 1988, a WA inspection of narrow-leafed lupin crops and field experiments also found the non-necrotic phenotype (Figure 2B and Figure 4G–J) [53,59,63,64]. In 1997, an inspection of 1000 plants at the edges of each of 102 narrow-leafed lupin crops growing in WA found plants with typical symptoms of the necrotic or non-necrotic BYMV strains in 100 and 64 crops, respectively. The necrotic strain sometimes occurred alone, but the non-necrotic strain only occurred when the necrotic strain was also present [59]. At individual sites, the incidences of plants with each symptom type were 0.1 to 7% (non-necrotic strain) and 0.3 to 56% (necrotic strain). When 34 wild narrow-leafed lupin populations were examined, 31 (91%) and 9 (26%) of them had plants with necrotic strain symptoms alone or both types of symptoms, respectively, with the incidences of plants with symptoms being 0.3–7% (non-necrotic strain) and 0.1–28% (necrotic strain). Again, although plants with non-necrotic symptoms always occurred amongst plants with necrotic symptoms, plants with necrotic symptoms sometimes occurred alone. When either strain was inoculated to plants of other lupin species, they both caused only non-necrotic symptoms. Moreover, both were stable as a repeated subculture for 3.5 years in subterranean clover plants did not alter the symptoms that each strain caused when inoculated to narrow-leafed lupin plants. As the non-necrotic strain never occurred in the absence of the necrotic strain and always infected fewer plants in these surveys, it may be less competitive when infecting subterranean clover plants within the infected pastures from which aphids spread BYMV to nearby lupin crops [59]. Since the necrotic phenotype in narrow-leafed lupin occurs in the presence of a BYMV-hypersensitive resistance gene (Section 5.4 below), such reversion to a wild type is consistent with the tendency of resistance-breaking virus strains to lack fitness when multiplying within plants lacking resistance genes [65,66].

In 1989, when seeds were harvested from single, naturally infected narrow-leafed lupin cv. Gungurru plants without symptoms or with systemic necrotic symptoms caused by BYMV infection, a yield loss of 94% was recorded [53]. This overall yield loss was not 100% because, following mature plant infection, the virus does not reach all terminal branches, whereas early infection results in all plant parts being invaded. In 1997, when the yields of naturally infected narrow-leafed lupin cv. Merrit plants with severe non-necrotic symptoms were compared with those of healthy plants, seed yield losses of 95% were recorded [59]. A later study found that seed yield losses in individual, naturally infected narrow-leafed lupin plants depended upon the stage of growth in which plants became infected. Thus, when BYMV infection was later, the seed yield losses were 56% (non-necrotic symptoms) and 84% (necrotic symptoms), whereas when BYMV infection was early, they were 99% (non-necrotic symptoms) and 100% (necrotic symptoms) [19,67]. With both BYMV strains, these yield losses arose from failure to produce seeds or producing fewer of them and from a reduction in seed size.

When seeds harvested from narrow-leafed plants with non-necrotic symptoms were tested for BYMV seed transmission to germinating radicles, no transmission was detected in radicles from 3190 or 3000 seeds of breeding line 90L423-07-13 or cv. Merrit, respectively, or radicles from 17,365 seeds of cv. Gungurru [68]. This lack of seed transmission with the non-necrotic BYMV strain in narrow-leafed lupin was unexpected as a lack of BYMV seed transmission with the necrotic strain had previously been attributed to the severe necrosis it causes. In contrast, low-level BYMV seed transmission of 0.2% was detected in tests on radicles of 10,130 seeds from BYMV-infected yellow lupin cv. Wodjil plants. More recently, when seed samples from BYMV-infected white lupin crops growing in northern NSW were tested in 2020 and 2021, seed transmission rates of 0.8–5% and 2%, respectively, were obtained [69].

### 5.2. Black Pod Syndrome

The first record of BPS affecting narrow-leafed lupin crops was in WA in the 1990s [60]. BPS appears relatively late in the growth of lupin plants and consists of flat back pods containing no or very few seeds (Figure 4F). When many plants develop BPS within a crop, seed yields are decreased considerably, with losses of 40% being recorded [70]. Cultivars differed in the rate at which BPS developed. For example, BPS was slow to appear in Jenabillup, but quick to appear in cv. Mandelup, causing greater yield losses in the latter. Buirchell [70] interpreted this difference as Jenabillup being less ‘susceptible’ to BPS. There was much debate over what was responsible for it. Three possible causes were hypothesized to explain BPS: (i) too much vegetative growth of foliage resulting in blackened pods, (ii) nutrient deficiencies having the same effect and (iii) pod symptoms caused by late BYMV infection. In BYMV-prone districts, studies involving extensive field experimentation with added growth hormone or fertilizer treatments failed to find evidence supporting hypotheses (i) and (ii) [71]. However, both annual ratings for BPS in 1996–2003 annual crop variety trials (CVT’s) and findings from field experiments in which frequent, regular high rates of foliar insecticide sprays were applied to kill aphid vectors [60,62] suggested possible BYMV involvement. This was despite BPS sometimes occurring in the primary pods of narrow-leafed lupin plants without any of the necrotic stem streaking that normally develops when BYMV infects them [3] or any BYMV being detected by ELISA in leaf samples.

In 2001, naturally BPS-affected narrow-leafed lupin plants were obtained from six sites in WA and tissue samples taken [45]. When these samples were tested individually by RT-PCR and ELISA, BYMV detection was most reliable when generic potyvirus virus primers were used in RT-PCR tests on samples of main stem tissue taken just underneath the blackened pods [45]. In two glasshouse experiments, BYMV isolates MI (Section 4 above) (exp 1) or AR93C obtained from a narrow-leafed lupin plant with BPS (exp 2) were cultured in subterranean clover plants. Infective sap from these cultures was inoculated to healthy narrow-leafed lupin cv. Mandelup and cv. Jenabillup plants at different growth stages in exps 1 and 2. BPS only appeared following inoculation at the growth stage when pods developed after first flowering in exp 1 and at this and the three later growth stages, but not in the four beforehand in exp 2. Inoculation before this growth stage always elicited systemic necrosis followed by plant death. In both experiments, although the BPS developed more slowly in plants of cv. Jenabillup than cv. Mandelup, when fully developed, its appearance was the same regardless of which cultivar was affected. Whenever BPS was induced by BYMV inoculation, testing samples by ELISA or RT-PCR confirmed its presence. Therefore, this study satisfied all four conditions of Koch’s postulates required to confirm that BPS is elicited by late BYMV infection. Condition 1—“That the pathogen is regularly associated with the disease”—was fulfilled by the findings at the six survey sites. Condition 2—“That the pathogen should be isolated from diseased plants and grown in culture”—was fulfilled by culturing it in subterranean clover hosts. Condition 3—“That the disease should be reproduced when a culture of the pathogen is used to introduce disease to a healthy susceptible host”—was fulfilled by the two glasshouse experiments that studied the effect of the plant growth stage at the time of inoculation. Condition 4—“That the pathogen should be detected in or reisolated from the inoculated plants”—was fulfilled by detecting BYMV in samples from inoculated plants that developed BPS [45].

In host range studies, seven BYMV isolates and one CYVV isolate were sap-inoculated to young plants belonging to three narrow-leafed lupin genotypes (cv. Jenabillup, cv. Mandelup and accession no. P26697) and to single genotypes of subterranean clover, field pea, faba bean, *Chenopodium quinoa* and *C. amaranticolor* [46]. These seven BYMV isolates were originally from narrow-leafed lupin plants with systemic necrosis (3), BPS (1) or non-necrotic symptoms (1) or from infected plants of sandplain lupin (1) or *L. pilosus* (1). Narrow-leafed lupin cvs Jenabillup and Mandelup were included because BPS symptoms develop at different rates in them [70], and accession P26697 because it carries a second narrow-leafed lupin hypersensitive specificity (Section 5.4 below). There were no differences between host species phenotypes attributable to BYMV infection that would be suitable for use in distinguishing isolates from narrow-leafed lupin plants with BPS from those with typical necrotic or non-necrotic symptoms. Amongst the isolates that provided complete datasets and were originally from narrow-leafed lupin, one from a plant with BPS and one from a plant with systemic necrosis, both caused systemic necrosis in Mandelup and Jenabillup and the CYVV isolate behaved similarly. Also, no differences in the rates of systemic necrotic symptom development were evident between the Mandelup and Jenabillup plants inoculated with these two BYMV isolates. A second BYMV isolate from a plant with systemic necrosis and an isolate from a plant with non-necrotic symptoms both caused non-necrotic symptoms in Mandelup and Jenabillup, and the isolate from sandplain lupin behaved similarly. Amongst the four BYMV isolates that infected P26697, only the one from *L. pilosus* caused systemic necrosis in this genotype, with the other three all causing non-necrotic phenotypes [46]. Therefore, although cv. Mandelup was considered more BPS-‘susceptible’ than cv. Jenabillup in field situations [70], the glasshouse studies of Kehoe et al. [46] did not confirm this suggestion because final BPS symptoms and their severity were the same in both cultivars despite developing faster in Mandelup than Jenabillup plants inoculated simultaneously. Nevertheless, since the delayed BPS development in the Jenabillup trait could still be important in future breeding for resistance to BPS in narrow-leafed lupin, knowledge of its likely nature (resistance to systemic infection via the phloem or mature plant resistance, etc.) and its genetic basis (likely to be polygenic) deserves further investigation.

### 5.3. Cultural Control

**Figure 5:**
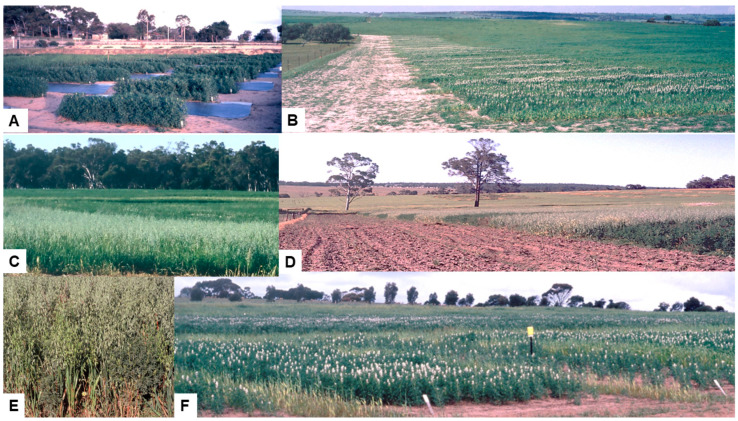
Images of cultural control experiments examining the effects of reflective mulch, non-host barriers, admixture with a non-host and different plant densities upon spread of bean yellow mosaic virus (BYMV) necrotic strain from an external source into narrow-leafed lupin (*Lupinus angustifolius*) stands. (**A**) Small-scale field experiment evaluating the effectiveness of reflective mulch in reducing BYMV and cucumber mosaic virus spread into single-row plots of narrow-leafed lupin by reducing aphid landing rates (at South Perth in 1988). (**B**) Typical design of large-scale field experiments used to study the effectiveness of different cultural control measures at reducing BYMV spread into narrow-leafed lupin crops from a nearby virus source. The plots were arranged in single file at the crop edge and were separated from the pasture by a cultivated fallow strip (at Badgingarra in 1994). (**C**) Non-host oat barrier plots of 30 m long × 15 m wide trip (front) that diminished BYMV spread to a narrow-leafed lupin cv. Danja crop (behind). These were alternated with fallow plots 30 m long × 15 m wide (not shown are images of fallow plots that failed to diminish BYMV ingress into the crop) (at Narrogin in 1987). (**D**) Design of large-scale field experiment used to study admixture with a non-host (oats) upon the spread of BYMV into lupin crop (behind). Plots of each type were alternated and numbers of BYMV-affected lupin plants were counted within a central 10 m × 10 m square within each plot (at Mount Barker in 1989). (**E**) Appearance of plants within an oat–lupin admixture plot (at Mount Barker in 1989). (**F**) Example of a field experiment examining the effect of plant density upon the spread of BYMV into narrow-leafed lupin plots arranged in a single file at the crop edge. Plots were sown at seedling rates of 25, 50, 75, 100 and 125 kg/ha in a randomized block design and were separated by a 1.5 m wide non-host (wheat) buffer (at Avondale in 1991).

Three field experiments in WA in 1987–1989 (1/year) studied whether surrounding single-row plots of narrow-leafed lupin with reflective mulch would diminish aphid landings, thereby decreasing CMV spread [72]. In addition to recording the spread from CMV infection sources to healthy lupins, the spread of necrotic strain BYMV into the same single-row plots was also recorded by counting the numbers of plants with typical necrotic symptoms (Figure 5A). In 1987, the external source for this spread was a nearby BYMV-infected block of lupin breeding lines (mostly of *L. pilosus*), but in 1988–1989, it remained unidentified. When representative leaf samples from lupin plants with BYMV symptoms were tested, this always confirmed its presence. In 1987 and 1988, the presence of reflective mulch markedly decreased the numbers of BYMV-infected plants, but in 1989, BYMV spread occurred later so the reduction in the spread to mulch-protected plots was less. As with CMV, the Australian narrow-leafed lupin breeding program was recommended to help protect its single-row plots from BYMV infection using reflective mulch.

**Figure 6:**
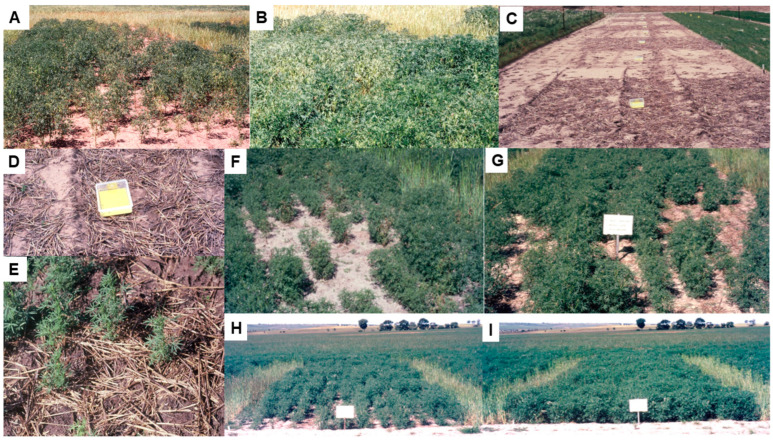
Images of cultural control experiments examining the effects of different plant densities (1990–1991) or plant densities, row spacings and stubble retention (1992), upon spread of bean yellow mosaic virus (BYMV) necrotic strain from an external infected subterranean clover pasture source into narrow-leafed lupin (*Lupinus angustifolius*) stands. (**A**) Appearance at end of growing season of plot originally sown at the low seeding rate of 25 kg/ha in which many plants had been killed or were being killed by infection with BYMV necrotic strain (due the lack of any groundcover or canopy cover that would reduce aphid vector landings) (at Avondale in 1991). (**B**) Appearance at end of growing season of plot originally sown at the high seeding rate of 125 kg/ha lacking visible damage from BYMV infection (because the high plant density prior to canopy closure and resulting rapid canopy development had helped repel incoming aphid vector landings) (at Avondale in 1991). (**C**) Small-scale field experiment in which centrally placed yellow water traps caught aphids flying over plots with or without straw added at 2 t/ha (at South Perth in 1992). (**D**) Close up of soil surface within the small-scale field experiment showing yellow water trap placement and distribution of added straw (at South Perth in 1992). (**E**) Close up of soil surface showing distribution of added straw within a plot with recently germinated lupin seedlings from a field experiment examining the effects of straw mulch (2 t/ha), row spacing and plant density upon BYMV spread; plot sown at low seeding rate of 30 kg/ha and narrow row spacing of 17.5 cm (at Badgingarra in 1992). (**F**) Appearance of plot without added straw originally sown at the low seeding rate of 30 kg/ha with wide row spacing of 35 cm with many plants being killed by infection with necrotic BYMV (due lack of sufficient groundcover or adequate canopy cover that would reduce aphid vector landings) (at Badgingarra in 1992). (**G**) Appearance of plot with added straw at 2 t/ha originally sown at the low seeding rate of 30 kg/ha with wide row spacing of 35 cm in which fewer plants were being killed by infection with necrotic BYMV (due to reduced aphid vector landings resulting from straw groundcover presence) (at Badgingarra in 1992). (**H**) Appearance of plot with straw mulch added at 2 t/ha originally sown at the normal seeding rate of 60 kg/ha with wide row spacing of 35 cm in which few plants were being killed by infection with necrotic BYMV (due to reduced aphid vector landings resulting from presence of straw groundcover and the greater plant density within rows) (at Badgingarra in 1992). (**I**) Appearance of reference plot without added straw originally sown at the high seeding rate of 100 kg/ha with narrow row spacing of 17.5 cm in which very few plants were killed by infection with necrotic BYMV (due to reduced aphid vector landings resulting from greater plant density within rows and rapid plant canopy development covering the bare ground in between narrowly spaced rows) (at Badgingarra in 1992).

In 1987–1991 in WA, field experimentation was used at four different sites to study the effects of three different cultural control measures upon the BYMV spread from an adjacent pasture into narrow-leafed lupin stands [53]. These and later cultural control experiments were each planted as a single file of plots separated by a fallow unplanted strip of soil from an adjacent subterranean clover pasture which acted as the source of BYMV (Figure 5B). Naturally occurring aphid vectors spread the virus from this source to the lupin plots. Wherever possible, the rows of plots were situated at the windward edges of lupin fields. The 1987–1991 experiments compared (i) a non-BYMV host (wheat or oat) versus fallow borders (1987–1988, three exps, three sites) (Figure 5C); (ii) admixture with a non-host (oats) versus a monoculture lupin (1988–1989, three exps, three sites) (Figure 5D,E); and (iii) varying lupin plant densities (1990–1991, three exps, two sites) (Figure 5F and Figure 6A,B) [53].

In 1987, a single perimeter file of alternating fallow or oat plots 30 m long × 15 m wide separated the BYMV infection source in adjacent subterranean clover pasture from a commercial lupin cv. Danja crop (Figure 5C) [53]. In 1988, along the field edge adjacent to subterranean clover pasture, each plot within a single file of 10 × 10 m square lupin cv. Danja plots was surrounded by alternating 15 m wide fallow or non-host borders; the non-host was wheat at one site but oats at the other. At final assessment, the presence of a ‘virus cleansing’ non-host border diminished mean numbers of lupin plants with necrotic BYMV by 51–53%. Taking the plant density into account, the percentage of affected plants was reduced by 43–60%. (Note: the term ‘virus cleansing’ is used here because when an aphid vector spreading a virus non-persistently lands on and probes a non-host plant species whilst searching for its preferred host(s), virus particles become detached from its stylet, rendering it poorly infectious.) This prevents a high incidence of infection from appearing at the crop margin. In 1988, at a small-scale field experiment site with a history of BYMV spread, miniature 1.5 × 1.5 m square plots sown as lupin cv. Danja/oat mixtures or lupin cv. Danja alone were separated from each other by 1 m of fallow. In 1989, at two sites, a single file consisting of alternating 15 × 15 m square plots of lupin cv. Danja/oats or cv. Danja alone was sown adjacent to subterranean clover pasture (Figure 5D,E). At final assessment, the number of lupin plants with necrotic BYMV symptoms was diminished by 76–96% in plots containing mixtures of lupin and oats. In 1990–1991, single files of 4.5 × 15 m lupin cv. Danja plots were sown adjacent to subterranean clover pasture at sowing rates of 20 (8.3–8.9), 50 (18.1–20.0), 80 (26.9–30.0) and 110 (35.8–36.3) kg/ha (resulting plants/m^2^) at two sites in 1990, but at 25 (11.0), 50 (20.1), 75 (28.4), 100 (35.3) and 125 (42.8) kg/ha (resulting plants/m^2^) at a single site in 1991 (Figure 5F). A non-host cereal barrier 1.5 m wide of oats (in 1990) or wheat (in 1991) separated each plot and a randomized block design was used. In the two experiments in which the plants grew well and a lupin plant canopy formed, the incidence of plants with necrotic BYMV was greatest at the lower and least at the higher plant densities regardless of whether the edge plants were included or excluded (Figure 6A,B). For example, in the 1990 experiment (in which the lupin plants grew well) at plant densities of 8.3, 18.1, 26.9 and 35.8 plants/m^2^, the numbers of plants with necrotic BYMV symptoms at final assessment (figures excluding edge plants in parentheses) were 69 (46), 42 (30), 39 (26) and 34 (23), respectively. Moreover, the smaller proportion of plants infected at higher plant densities was not just from the greater lupin plant numbers/m^2^ as this proportion still declined when adjustments took the plant numbers present into account. Therefore, the greater BYMV incidence in sparse stands was due not only to the smaller plant numbers, but also to a second factor, namely incoming infective vector aphids being more attracted to plants with bare earth around them than to plots with a dense canopy. This second factor is the ‘bare earth effect’ reported previously with other pathosystems [73,74]. In the 1990 experiment, the grain yield loss diminished as the plant density increased. The greatest yield loss was about one third, which arose at the lowest plant density when 34% of plants had necrotic symptoms [53]. Correcting them to take into account the plants killed increased the losses proportionately less as the lupin plant densities increased. In 1990–1991, the main aphid vector species trapped near these field experiments were *A. craccivora*, *Acyrthosiphon kondoi*, *Myzus persicae*, *Lipaphis pseudobrassicae* and *R. padi*. In addition, small numbers of *Acyrthosiphon pisum*, *R. maidis*, *R. insertum* and *R. rufiabdominalis* were sometimes also trapped. The principal BYMV vector species were *Acyrthosiphon kondoi* and *Myzus persicae*. These field experiments revealed that sowing a non-host cereal barrier around its margin can diminish the BYMV spread from an external source into a narrow-leafed lupin crop and sowing at high seeding rates to generate high plant densities and early canopy closure can reduce the BYMV spread within such crops. They also showed that mixed cropping with narrow-leafed lupin and a non-host cereal can reduce the BYMV spread. Unlike sowing a non-host barrier and sowing at high seedling rates to increase seed yields, the main benefit from adopting a mixed cropping approach would be to help produce protein-rich hay [53].

In 1992, identical field experiments sown at two WA sites (exps 1 and 2) studied whether simulated stubble retention (=direct drilling into cereal stubble) influences the BYMV spread into narrow-leafed lupin stands sown at different seeding rates and row spacings [64]. Single files of rectangular 4.2 m wide × 15 m long plots were sown at the windward edge of a field adjacent to a subterranean clover pasture BYMV source. A randomized block design with four replications and nine treatments that consisted of all possible combinations of 2 t/ha straw added to the soil surface after seeding or no straw and narrow (17.5 cm) or wide (35 cm) row spacing at low (25–30 kg/ha) or normal (50–60 kg/ha) seeding rates. A ‘reference treatment’ that reflected the high seeding rate (90–100 kg/ha), narrow row spacing and lack of stubble retention recommended commercially at that time was also included. On six dates during the growing period, the numbers of plants with necrotic BYMV symptoms/plot were recorded and the percentage of plants affected/plot calculated. On each date, the lupin plots were also inspected for aphid colonies, but none were found in either experiment. Aphid traps associated with exp 2 mostly caught alates of *Myzus persicae* and *R. padi*, but a few *Acyrthosiphon kondoi* and cereal root aphids (*R. rufiabdominalis* or *R. insertum*) were also caught. Moreover, in a related small-scale experiment in 1992, when flying aphids were caught in water traps placed within plots with or without straw added at 2 t/ha (Figure 6C,D), the numbers of *Myzus persicae* caught in the absence of straw were significantly greater than in its presence. In contrast, there were no significant differences with *R. padi* or *A. kondoi*. In exp 1, the BYMV spread was substantial, but in exp 2, it was smaller. Nevertheless, in both, the presence of added straw (Figure 6E) decreased the BYMV incidence significantly, and this occurred regardless of whether the row spacing was wide or narrow or the plots were sown at medium or low seeding rates. At the final assessment, this decrease was 72–75% in exp 1 and 25–27% in exp 2 (Figure 6F–H). This reduction in the BYMV spread in the presence of straw was due to an absence of the ‘bare earth effect’ (see previous paragraph), so landings of incoming aphid vector alatae landings were diminished. In exp 1, but not in exp 2, a separate analysis of the data from treatments without straw mulch revealed that narrow row spacing and sowing at the normal seeding rate also decreased the BYMV spread significantly, by 38% for narrow spacing and 48% for the normal seeding rate. This row spacing decrease was attributable to diminished aphid vector landings arising from the faster canopy closure across narrowly spaced rows, whereas the normal seeding rate decrease arose from the combination of greater plant numbers and more rapid canopy development in reducing aphid vector landings. The early development of a dense canopy generated by the ‘reference treatment’ also diminished early BYMV spread in both experiments, but accurate BYMV counts were impossible later due to the canopy becoming too dense. There were significant effects of straw mulch being present upon seed yields in exp 1, with these yields being increased by 27% and 16% at low and normal seeding rates, respectively. However, there were no significant yield differences attributable to narrow versus wide row spacing. The yield from the ‘reference treatment’ (Figure 6I) was not significantly greater than those of both added straw treatments sown at the normal seeding rate (i.e., with row spacing that was wide or narrow) [64]. These findings suggest that direct drilling into cereal stubble (simulated here by adding straw mulch to the soil surface) can be recommended to help supress yield losses from BYMV infection in narrow-leafed lupin crops. They also suggest that, because of the greater BYMV spread that results, using wide row spacing in the absence of stubble is undesirable in regions where subterranean clover pastures are widespread.

### 5.4. Host Resistance

**Figure 7:**
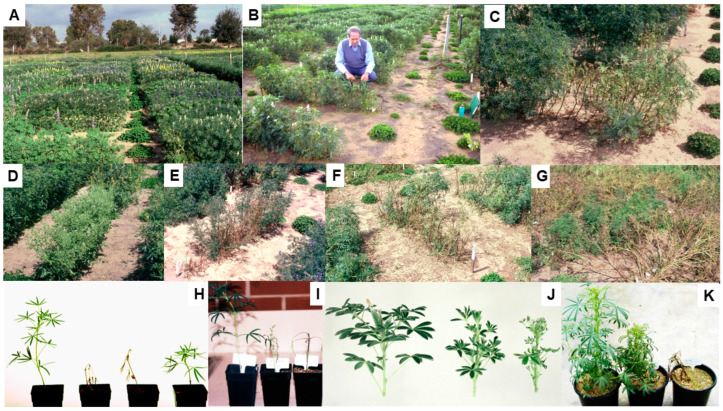
Images of bean yellow mosaic virus (BYMV) symptoms from host resistance studies with narrow-leafed lupin (*L. angustifolius*) and other lupin species. (**A**) Annual lupin BYMV resistance screening employing single row plots containing cultivars, breeding lines and germplasm accessions of different lupin species. Subterranean clover transplants infected with BYMV necrotic strain isolate MI placed at both ends of each plot to ensure a uniform inoculum source for spread by naturally occurring aphids to the lupin rows (at South Perth in 1993). (**B**) Recording spread of BYMV infection in annual lupin BYMV resistance screening focused on cultivars, breeding lines and germplasm accessions of narrow-leafed lupin. Note smaller BYMV-infected subterranean clover virus source plants at both ends of each row with larger initially healthy source subterranean clover source plants in between each of them that, once infected, increased virus source potency for spread to the lupin rows (at South Perth in 1997). (**C**) BYMV necrotic strain spread within a plot of narrow-leafed lupin causing systemic necrotic symptoms at different stages of development in infected plants (at South Perth in 1992). (**D**) BYMV necrotic strain spread along an entire single-row plot of sandplain lupin (*L. cosentinii*) cv. Eragulla causing non-necrotic symptoms of severe leaf mosaic, deformation, reduction in size and plant stunting (at South Perth in 1995). (**E**) BYMV necrotic strain spreading within a plot of narrow-leafed lupin causing systemic necrotic symptoms at different stages of development within infected plants (at South Perth in 1992). (**F**) BYMV necrotic strain spreading within a plot of narrow-leafed lupin causing systemic necrotic symptoms at different stages of development within infected plants (at South Perth in 1997). (**G**) BYMV necrotic strain symptoms within a plot of narrow-leafed lupin germplasm accession P26697 in which, instead of causing necrotic symptoms, this strain elicited non-necrotic symptoms consisting of systemic mosaic and leaf deformation (central row) contrasting with rows of other accessions in which the plants were killed (rows in front in bottom right position and behind in top left position) (at South Perth in 1995). (**H**) Aphid transmissions with BYMV to plants of narrow-leafed lupin cv. Danja, uninoculated control plant (left), necrotic isolates MI (center left) and LP (center right) causing systemic necrosis and plant death, and non-necrotic isolate LKtg2-NN causing upright habit, decreased leaf size and stunting (right). (**I**) Aphid transmissions with BYMV to plants of narrow-leafed lupin accession number P26697, uninoculated control plant (left), necrotic strain isolate MI (center) causing upright habit, decreased leaf size and stunting, necrotic strain isolate LP (right) causing the necrotic symptoms systemic necrosis and plant death. (**J**) Aphid transmissions with BYMV to plants of white lupin (*L. albus*) cv. Kiev Mutant: uninoculated control plant (left), non-necrotic strain isolate LKtg1-NN (center) causing mild upright habit and decrease in leaf size, and non-necrotic strain isolate LEsp-NN (right) causing severe upright habit, pallor and decrease in leaf size. (**K**) Aphid transmissions to F2 narrow-leafed lupin progeny plants from the cross P26697 × Merrit demonstrating non-necrotic plant dwarfing (center) and plant death (right) caused by necrotic BYMV strain isolate MI; healthy plant (left).

The routine field screening procedure developed in 1989–1990 to evaluate different legume genotypes for their responses to BYMV-isolate MI infection (Section 4 above) was used annually thereafter for routine screening for BYMV resistance by the Australian national lupin breeding program (Figure 7A–D) [16,24,61,75]. Irrigation was used to ensure the plants grew well and extend the growing period, and the plants were thinned so there was always a uniform number of plants present/row. Apart from just two exceptions, every year, infected plants of narrow-leafed lupin cultivars, breeding lines and germplasm accessions always developed a systemic necrotic phenotype (Figure 7C,E,F) [61,75]. The two exceptions were narrow-leafed lupin breeding line 90L423-07-13 and accession no. P26697, both of which always developed a non-necrotic phenotype (Figure 7G). When 51 BYMV isolates obtained in WA during 1988–1998 from 10 different species (narrow-leafed, yellow, white or sandplain lupin, *L. pilosus*, faba bean, subterranean clover, *Melilotus indica*, *Kennedia prostrata* or *Gladiolus*) were each aphid-transmitted using *Myzus persicae* from cultures maintained in subterranean clover plants to plants of different genotypes of narrow-leafed lupin, the phenotypes that developed were either necrotic or non-necrotic [47]. When each isolate was inoculated to narrow-leafed lupin cv. Danja, 10 and 41 isolates elicited necrotic or non-necrotic BYMV phenotypes, respectively (Figure 7H). Further, when 37 of the 41 isolates that elicited non-necrotic phenotypes were inoculated to breeding line 90L423-07-13, all of them still caused non-necrotic phenotypes. In contrast, when nine of the ten isolates that caused necrotic phenotypes in cv. Danja were inoculated to breeding line 90L423-07-13, only two of them (LP and SV) still elicited necrotic phenotypes, with the other seven eliciting non-necrotic symptoms. Moreover, when some of these BYMV isolates were inoculated to accession no. P26697 and cv. Merrit, the phenotypes that resulted always resembled those obtained in 90L423-07-13 or Danja, respectively (Figure 7I). Thus, the narrow-leafed lupin genotypes inoculated exhibited one of two strain-specific, systemic-hypersensitive (=necrotic) specificities in response to infection with different BYMV isolates. This finding suggested that four different biological strain groups (=pathotypes) of BYMV were present, with Group 1 being represented by the two isolates (LP and SV) that elicited both hypersensitive specificities (e.g., isolate LP in Figure 7H,I); Group 2 by seven isolates that elicited the specificity in Danja but not in 90L423-07-13 or P26697 (e.g., isolate MI Figure 7H,I); Group 3 by hypothetical isolates that elicit the specificity in 90L423-07-13 or P26697 but not the one in Danja; and; Group 4 by the 37 isolates that failed to elicit either specificity (e.g., isolate LKtg2-NN in Figure 7H) [47]. None of these four strain groups were evident when BYMV isolates were inoculated to other lupin species, although their non-necrotic symptoms differed in virulence (e.g., white lupin (*L. albus*) in Figure 7J). Cross protection against a necrosis-inducing isolate was exhibited when the narrow-leafed lupin plants inoculated with it were already infected with an isolate that failed to elicit necrosis. Moreover, when one isolate of each type were both inoculated together to subterranean clover plants and left to multiply in a mixed infection for nine months, the necrotic isolate outcompeted the non-necrotic isolate so that it was no longer detectable. These findings are consistent with the non-necrotic BYMV strain being less fit than the necrotic strain when both infect the same subterranean clover plant. However, this apparent lack of fitness was not due to differences in aphid transmissibility as non-necrotic and necrotic isolates were transmitted at similar efficiencies by *Myzus persicae* [47].

Cheng and Jones [47] concluded that the two systemic hypersensitive specificities revealed when different narrow-leafed lupin genotypes were responding to infection with isolates belonging to different BYMV strain groups might arise from the presence of two independently inherited strain-specific hypersensitivity genes. To explore this further, BYMV isolate MI was inoculated to the F2 progeny plants from six crosses, the parents of which were the genotypes 90L423-07-13 and P26697, which respond to infection without necrosis, and cvs Danja and Merrit, which respond to infection with necrosis [76]. These six crosses were 90L423-07-13 × Danja, 90L423-07-13 × Merrit, P26697 × Danja, P26697 × Merrit, 90L423-07-13 × P26697 and Danja × Merrit. In the four crosses between non-necrotically and necrotically reacting genotypes, a 3:1 segregation ratio (necrotic: non-necrotic) was always obtained in the F2 progeny plants (e.g., with the cross P26697 × Merrit; see Figure 7K). In contrast, 99% of the F2 progeny plants from the cross between the two cultivars developed necrosis, whereas only non-necrotic phenotypes developed in F2 progeny plants from the cross between the two breeding lines. These findings suggested that the systemic necrotic phenotype that formed following the inoculation of F2 plants with BYMV isolate MI is controlled by a single dominant hypersensitivity gene, which they named *Nbm-1*. However, independently segregating modifier genes that altered symptom expression were apparently operating in the genetic background. This was suggested by the delay between inoculation and being killed varying markedly from plant to plant, with the progeny plants from crosses involving parents responding with and without necrosis, especially when accession P26697 was a parent [76]. Since isolate MI belongs to BYMV strain group 2, the inoculation of F2 progeny plants from the same crosses with an isolate belonging to BYMV strain group 3 would be required to provide evidence for the presence of the proposed second independently inherited strain-specific hypersensitivity gene. Since all narrow-leafed lupin cultivars already contain *Nbm-1*, lupin breeding programs would not need to check for its presence when producing new cultivars unless they use parents like 90L423-07-13 or P26697 for their crosses. Systemic hypersensitivity limits virus spread in the field by operating at the plant population level rather than at individual plant level. Thus, despite its drastic impact on the yields of individual infected plants, the rapid death of early-infected plants removes them as a source of virus infection for further spread by aphids, thereby greatly diminishing the spread of necrotic BYMV (Section 5.5 below) [53,68,75,77,78,79,80,81].

Partial resistance to BYMV infection by naturally occurring aphid vectors, known as ‘infection resistance’, was also found during annual routine screening for BYMV resistance in narrow-leafed lupin breeding lines and germplasm accessions [24,61]. Different genotypes growing in single-row plots exposed to a uniform BYMV inoculum pressure from having BYMV-isolate MI-infected subterranean clover transplants positioned at each of their row ends developed a spectrum of responses varying from being ‘highly susceptible’ through to having ‘infection resistance’. This type of partial resistance was not related to the alkaloid content, aphid susceptibility or flowering date, but associated with requiring many more infectious vector aphids to establish BYMV infection successfully [24,61]. It is likely to be a quantitatively inherited trait. The highest level of BYMV ‘infection resistance’ located was in breeding line 84A086-5-20-31 [61], which was confirmed in narrow-leafed lupin breeding trials where the BYMV spread occurred naturally [60]. Breeding line 84A086-5-20-31 was, therefore, recommended for use as a parent in crosses aimed at producing new lupin cultivars suitable for growing in BYMV-prone high-rainfall regions where abundant BYMV infection reservoirs and smaller-sized fields growing in close proximity to BYMV-infected subterranean clover pastures cause many plants to die in years where aphid vector arrival occurs early in the growing season [16,20,75,77,78].

During 1993–2007, Australian studies examined the feasibility of employing genetic engineering to introduce BYMV resistance to plants of narrow-leafed and yellow lupin [16,75,82,83,84,85,86]. The principal approach adopted involved incorporating constructs of the viral protease (NIa) gene from BYMV isolate MI. Six different NIa constructs were introduced to four cultivars each of narrow-leafed and yellow lupin. In 1997–1998, when transgenic progeny plants belonging to 25 yellow lupin (T3-T6 generations) and 11 narrow-leafed lupin (T2–T3 generations) lines were inoculated with BYMV, most were susceptible, but 12 yellow lupin and 2 narrow-leafed lupin lines contained a few plants that never became infected [83]. Subsequently, however, when further transgenic progeny plants with the NIa gene constructs belonging to 31 yellow lupin and 26 narrow-leafed lupin lines were inoculated with BYMV isolate MI, no extreme resistance was found. However, 11/310 yellow lupin plants containing two different NIa constructs showed partial resistance consisting of delayed systemic movement. This partial resistance was absent from the 249 transgenic narrow-leafed lupin plants tested [84]. In addition, the possibility of conferring BYMV resistance using a synthetic ‘hairpin’ construct derived from the replicase (NIb) gene of isolate MI was studied in plants of narrow-leafed lupin cv. Wonga [86]. When the progeny plants of 45 lines with this ‘hairpin’ construct were challenged by repeated inoculation with BYMV over two generations (T2–T3), seven lines that had extreme resistance remained. These ‘immune’ plants each contained 1–2 copies of the ‘hairpin’ construct. By 2007, however, when further progeny plants from these transgenic plants were tested using five different BYMV isolates (including MI), this NIb gene construct-derived resistance had been silenced as its expression was absent regardless of the BYMV isolate [85]. As yet, there have been no genetic modification studies with BYMV in lupin using gene editing (CRISPR) or RNA silencing (RNAi) [87,88,89] to improve virus resistance in lupin.

### 5.5. Temporal and Spatial Patterns of Spread

When necrotic BYMV spread from adjacent infected subterranean clover pastures into the edges of narrow-leafed lupin crop or plots located at field edges (Figure 5B,D,F), the primary infection foci that incoming infectious vector apterae initiated consisted of single infected plants or, less often, pairs of infected plants [53,64]. These infected plants soon developed systemic necrosis and died. Occasionally, however, small patches of infected plants that represented limited secondary spread of infection developed around these initial infection foci. The reason for this secondary infection was that, during the brief time in between the initial systemic invasion of a lupin plant and necrosis becoming generalized, there is a brief period during which aphid vectors can acquire BYMV and spread it to other plants. Therefore, although the pattern of BYMV spread was predominantly monocyclic, a limited amount of secondary (i.e., polycyclic) spread also occurred [53]. In exp 1 of the two identical 1992 field experiments described above (Section 5.3), a similar pattern of isolated infected plants or pairs of plants, occasionally surrounded by small patches of infected plants, occurred within the plots with narrow row spacing without added straw [64]. However, in the plots with wide row spacing without added straw, there was limited spread along rows consisting of side-by-side sequences of up to 11 plants with systemic necrotic symptoms at different stages of symptom development. This suggested that aphid vector acquisition of BYMV within the brief period between initial systemic infection and generalized necrosis had occurred from plants infected at different times. In contrast, in the presence of added straw, the amount of BYMV spread was more limited and these patterns of virus spread were less evident regardless of whether rows were narrowly or widely spaced. In exp 2, in which there was less virus spread into the lupin plots, BYMV infection was limited to isolated infected plants or pairs of plants without any secondary spread [64].

**Figure 8:**
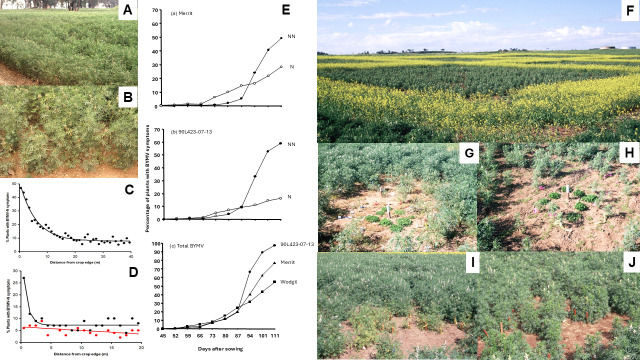
Images from studies on the temporal and spatial spread patterns of necrotic and non-necrotic bean yellow mosaic virus (BYMV) strains in narrow-leafed lupin (*Lupinus angustifolius*) (**A**–**D**,**F**–**J**) or both narrow-leafed and yellow (*L. luteus*) lupin (**E**). (**A**) Commercial crop of lupin cv. Merrit at site 1 into which BYMV necrotic strain was spread from an adjacent BYMV-infected subterranean clover pasture across a 10 m wide dirt track by naturally occurring aphid vectors (at The Lakes in 1993). (**B**) Close up of the cv. Merrit crop margin at site 1 showing high incidence of plants with necrotic BYMV symptoms moving from shoot apices to the rest of the infected plant (at The Lakes in 1993). (**C**) Gradient of plants symptomatic for BYMV necrotic strain in the cv. Merrit crop at site 1, starting at the crop edge closest to the BYMV-infected pasture source with fitted exponential line 7.76 + 43.7 (0.8406x) (at The Lakes in 1993). (**D**) Gradients of plants symptomatic for BYMV necrotic strain in a commercial lupin cv. Gungurru crop at site 2, starting from the crop edge bordering either a perimeter oat barrier 20 m wide that separated adjacent infected pasture from the crop with fitted linear line 6.561 − 0.1511x (red line) or internal tracks containing BYMV-infected clovers with fitted exponential line 7.308 + 37.7 (0.27x) (black line) (at West Dale in 1994). (**E**) Disease progress curves for BYMV comparing the spread of its necrotic (N) and non-necrotic (NN) strains in plants within replicated single-row (2.5 m) plots of cv. Merrit (a), breeding line 90L423-07-13 (b) or of both strains combined in Merrit, breeding line 90L423-07-13 or yellow lupin cv. Wodgil (c) (at South Perth in 1998). (**F**) Example of field experiment examining spread of necrotic and non-necrotic BYMV strains from introduced BYMV-infected subterranean clover (*Trifolium subterraneum*) infector plants within replicated cv. Gungurru lupin plots (17 × 18 m) arranged in a randomized block design and each surrounded by 5 m wide non-host canola (*Brassica napus*) buffers (at Avondale in 1998). (**G**) Early phase of BYMV spread by naturally occurring aphid vectors from a focus of subterranean clover transplants infected with necrotic strain isolate MI either killing or in the process of killing surrounding plants, each of which were tagged with different colored tapes; tape color indicated tagging date (exp 2, at Avondale in 1998). (**H**) Early phase of BYMV spread by naturally occurring aphid vectors from a focus of subterranean clover transplants infected with non-necrotic strain isolate LKoj1-NN causing apical chlorosis and stunting in surrounding plants, each of which were tagged with different colored tapes; tape color indicates tagging date (exp 2, at Avondale in 1998). (**I**) Intermediate phase of BYMV spread by naturally occurring aphid vectors causing plant death, or in the process of killing plants, growing in the vicinity of the original necrotic strain isolate MI infection focus; color-coded wooden stakes positioned next to each infected plant indicate tagging date (exp 3, at Avondale in 1999). (**J**) Intermediate phase of BYMV spread by naturally occurring aphid vectors causing leaf mosaic, chlorosis and size reduction and smaller sized plants growing in the vicinity of the original non-necrotic strain isolate LKoj1-NN infection focus; color-coded wooden stakes positioned next to each infected plant obscured by infected plant foliage growth (exp 3, at Avondale in 1999).

In 1993–1994, studies at two sites in WA examined the spatial patterns of BYMV spread in commercial narrow-leafed lupin crops [90]. In 1993, the crop of cv. Merrit studied was separated from a BYMV-infected subterranean clover pasture by a 15 m wide strip containing a 10 m wide dirt road (Figure 8A,B) (site 1). In 1994, a crop of cv. Gungurru was separated from a similar BYMV-infected pasture source by a 20 m wide strip of oats (site 2). Also in 1994, a 10.4 m × 15 m rectangular block sown with healthy cv. Gungurru seed was used to study BYMV spread (site 3) [90]. An 11 m wide narrow fallow strip combined with a perimeter oat barrier which was 25 cm wide along the northern edge of the lupin block separated it from an annual BYMV lupin resistance screening experiment (Section 5.4 above). This resistance screening experiment acted as a potent source of BYMV necrotic strain from which vector aphids spread the virus to healthy lupin plants within this block. At site 1, counts of the numbers of plants with necrotic BYMV symptoms were made inside square (1 × 1 m) quadrats along four transects 40 m long and 1 m wide starting at the crop margin and penetrating at right angles into it. At site 2, similar counts were made inside the same sized quadrats along eight transects 20 m long and 1 m wide. Four of these transects commenced at the boundary with the oat perimeter strip, but the other four commenced at internal vehicle tracks in which BYMV-infected subterranean clover plants were growing. At site 3, each individual plant was mapped and records about whether it was still healthy or had developed symptoms of BYMV necrotic strain infection were collected at regular intervals. The BYMV infection gradients from the crop edge at site 1 (Figure 8C) and from the internal vehicle tracks at site 2 (Figure 8D, black line) consisted of many plants with necrotic BYMV symptoms next to the crop edge, then a sharp decline in their numbers followed by a more gradual decline deeper into the crop. In contrast, there were no such gradients from the oat perimeter strip into the crop at site 2, just a very slow decline in incidence of symptomatic plants (Figure 8D, red line). At site 3, the gradient of symptomatic plants from the block edge closest to the source resembled the shallow gradient from the 20 m-wide oat perimeter strip into the crop at site 2. The explanation was that both the 20 m wide (site 2) and 25 cm wide (site 3) oat barriers were acting as ‘virus cleansing’ non-host buffers (Section 5.3 above) in between the virus source and the edge of the lupin crop.

At site 1, counts also examined the proximity of symptomatic plants to one another within square (2.8 × 2.8) quadrats placed at 5, 10, 20, 30 and 40 m along two transects from the crop edge. Although there were more single plant infections at 5 m, groups of 2, 3, 4–5, 6–10 and 11–20 symptomatic plants occurred in similar numbers, whereas at 10 m, the 11–20 plant category was lacking and pairs of infected plants were the most common. In contrast, at 20, 30 and 40 m, single symptomatic plants were the most common, followed by pairs, with very few symptomatic plants in any of the larger groups being present. Thus, at the crop edge, although many of the lupin plant infections were from incoming infective vector aphids, some were from non-infectious aphids that had acquired the virus from newly-infected lupin plants during the brief period while this was still possible before these plants developed necrosis and died. This enabled the aphids to spread it to 1–2 plants before they lost it again. In contrast, only the latter occurred deeper into the crop, hence the high frequency of infected plants occurring singly or in pairs [90].

Due to presence of a 25 cm wide non-host buffer, at site 3, it was also possible to study the secondary BYMV spread from an external source in a situation lacking interference from ‘edge effects’ [90]. Around the primary foci consisting of single or pairs of infected plants, the staggered spread of necrotic BYMV occurred in three phases. This took place along, and sometimes between, rows. The consequence of such a spread scenario was the development of side-by-side pairs, triples and occasionally quintuples of symptomatic plants. BYMV introduction to 1–2 plants over a distance by infectious aphid vectors constituted the first phase, its acquisition and spread by aphids to 1–2 more plants during the brief period before they developed necrosis and died constituted the second phase and the third phase arose from a second similar cycle of BYMV acquisition followed by transmission to 1–2 more plants [90]. These successive phases of spread provided evidence of the staggered infection pattern predicted by Thackray et al. [78]’s conceptual model for BYMV necrotic strain spread in narrow-leafed lupin stands. When counts for numbers of adjacent symptomatic plants were combined within 33.4 × 36 cm quadrats, the contour map obtained using these quadrat data revealed zones of small patch and gap clusters that intermingled, with patch clusters being more abundant closer to the BYMV source and gap clusters predominating further away. This pattern suggested diffuse spread causing small clusters, which is consistent with the shallow infection gradient starting at the block edge closest to the necrotic BYMV infection source. Both this pattern and the preponderance of single, isolated or paired symptomatic plants also suggested that new infections depended mostly on infectious aphid vectors flying from the external virus source rather than on the internal virus acquisition and spread within the lupin block [90].

In 1998–1999, the temporal patterns of BYMV spread that occur when necrotic or non-necrotic BYMV strains spread in narrow-leafed lupin stands were studied at three sites in four different situations [77]. The first situation involved a square 20 × 20 m recording area within a large, rectangular 60 × 40 m lupin cv. Gungurru block sown at 90 kg/ha, into which naturally occurring aphid vectors spread both types of BYMV strains from infected subterranean clover pasture growing at its windward edge. At frequent intervals, the plants that had recently developed each type of symptom were marked with color-coded stakes. The BYMV spread data obtained were used to develop disease progress curves for the percentages of plants with each symptom type. The spread of the necrotic strain followed a linear trajectory (monocyclic pattern). In contrast, although the non-necrotic strain arrived later, its curve accelerated upwards as time progressed (polycyclic pattern). Few aphids colonized the lupins; these were *Myzus persicae* predominantly, but occasionally *A. craccivora* or *Acyrthosiphon kondoi*. In the second situation (exp 1), single-row plots 2.5 m long were each sown with seeds of narrow-leafed lupin cvs Merrit, breeding line 90L423-07-13 or of yellow lupin cv. Wodjil. The plots of each lupin genotype treatment were replicated eight times. Each plot was surrounded by 1 m of bare earth and two non-host rows of oats surrounded the experiment. Subterranean clover transplants infected with non-necrotic strain isolates LutKP or LJKtg1-NN were placed at the ends of each row (a different isolate at each end). Each row was thinned to 150 plants. Naturally occurring aphid vectors spread necrotic strain BYMV into the experiment from nearby naturally occurring external sources, but spread non-necrotic BYMV internally from the introduced clover transplants at the ends of each row. In both narrow-leafed lupin genotypes, the disease progress curve for necrotic BYMV was almost linear (a near-monocyclic pattern), whereas the curve for non-necrotic BYMV accelerated upwards, infecting a significantly greater number of proportions of plants (polycyclic pattern) (Figure 8E(a,b)). Notably however, significantly fewer plants of 90L423-05-13 than of cv. Merrit developed necrotic symptoms. This suggested that isolates belonging to BYMV strain groups 1 and 2, rather than of strain group 1 on its own, were circulating at the experimental site used (South Perth). This deduction was because strain group 1 elicits a systemic necrotic phenotype with the hypersensitive specificities present in both lupin genotypes, whereas strain group 2 does so only with the hypersensitive specificity (gene *Nbm-1*) present in cv. Merrit (Section 5.4 above). Moreover, this scenario was what would be expected as isolates LP (strain group 1) and MI (strain group 2) both originally came from this same site. In cv. Wodjil, all the diseased plants developed non-necrotic BYMV symptoms reflecting the absence of systemic hypersensitivity to BYMV in yellow lupin and all three progress curves obtained accelerated upwards (Figure 8E(c)). No colonizing aphids were recorded [77].

**Figure 9:**
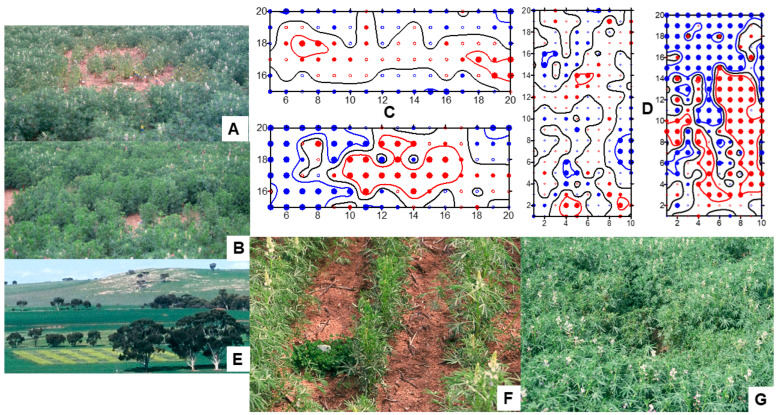
Images from studies on spatial patterns of spread of necrotic and non-necrotic bean yellow mosaic virus (BYMV) strains (**A**–**D**) or quantifying seed yield losses caused by its non-necrotic strain in narrow-leafed lupin (*Lupinus angustifolius*) (**E**–**G**). (**A**) Late phase of BYMV spread by naturally occurring aphid vectors causing plant death or in the process of killing lupin plants over a wider area around the original necrotic strain isolate MI infection focus; color-coded wooden stakes positioned next to each infected plant indicate tagging date (exp3, at Avondale in 1999). (**B**) Late phase of BYMV spread by naturally occurring aphid vectors causing leaf mosaic, chlorosis and size reduction and smaller sized plants affecting most of the lupin plants after spreading from the original non-necrotic strain isolate LKoj1-NN infection focus; color-coded wooden stakes positioned next to each infected plant now obscured by infected plant foliage growth, so late-infected plants are tagged with colored tape (exp 3, at Avondale in 1999). (**C**) Maps of clustering indices (v) for cumulative numbers of narrow-leafed lupin cv. Gungurru plants with BYMV symptoms at the ‘natural spread site’ in an area defined by (5 < x axis < 20, 15 < y axis < 20), comparing necrotic (270 infections) with non-necrotic (269 infections) BYMV at 137 days after sowing. Non-necrotic strain (map below) spread more widely than necrotic strain (map above). Axes show distances in meters and top axis, the boundary with adjacent BYMV-infected pasture. Red and blue spots and circles represent units denoting infection patches with ν > 0 (red) and infection gaps with ν < 0 (blue), respectively. Circles represent clustering indices of 0 to +/− 0.99 (clustering below expectation), small spots +/− 1 to +/− 1.49 (clustering exceeds expectation) and large spots > 1.5 or <−1.5 (half as much again as expectation). Red lines enclosing patch clusters are contours of ν = 1.5 and blue lines enclosing gap clusters are of ν = −1.5. Black lines are zero-value contours, representing boundaries between patch and gap regions where the count is close to the sample mean (natural spread site, at Mount Barker in 1998). (**D**) Maps of clustering indices at 144 days after sowing for cumulative numbers of narrow-leafed lupin cv. Gungurru plants with necrotic (left map) and non-necrotic (right map) BYMV symptoms in plot without introduced foci. Non-necrotic strain spread much more widely than necrotic strain. Symbols, contours and axes as for Figure 9C (exp 3, at Avondale in 1999). (**E**) Overview of field experiment with a randomized block design quantifying seed yield losses caused by BYMV non-necrotic strain in 5.6 × 15 m cv. Tanjil plots (green) surrounded by 5 m wide non-host canola buffers in flower (yellow) (at Avondale in 2001). (**F**) Initial BYMV spread from infected subterranean clover transplant to nearby plants in central row within plot of field experiment quantifying seed yield losses caused by BYMV non-necrotic strain (at Avondale in 2001). (**G**) Patch of shorter plants with non-necrotic BYMV symptoms (paler foliage, mostly not flowering yet) surrounding introduced BYMV infection focus within plot of field experiment quantifying seed yield losses caused by BYMV non-necrotic strain in narrow-leafed lupin (at Avondale in 2001).

In both the third (exp 2 in 1998)) and fourth (exp 3 in 1999) temporal spread pattern situations studied, the plots sown were sown with 25 kg/ha of narrow-leafed lupin cv. Gungurru seed and were almost square (17 × 18 m) (or rectangular (10 × 20 m), respectively [77]. A 5 m wide non-host barrier of canola surrounded each of them (Figure 8F). BYMV-infected infector transplants of subterranean clover were introduced to act as virus sources for spreading by naturally occurring aphids. In exp 2, there were three treatments each replicated twice: these were plots with introduced BYMV infector plants infected with necrotic (isolate MI) or non-necrotic (isolate LKoj1-NN) strains and control plots without infector plants. In plots with introduced infector plants, a 5 m diameter circular area around each of the five different introduced infector transplant foci within each plot (one central and four lateral) was marked initially with pegs and a rope. In exp 3, there were five treatments, each replicated four times: the same BYMV isolates were introduced to two sets of plots for each isolate and were subsequently removed earlier or later, providing two different treatments for each BYMV strain. The fifth treatment consisted of control plots without infector plants. Four infector transplants were introduced into each of the three infector transplant foci, which were arranged in a row along the center of the plot. In exp 2, few aphids colonized the lupins; *Myzus persicae* predominantly, but occasionally *A. craccivora* or *Acyrthosiphon kondoi*. In exp 3, the same three aphid species colonized the lupins, but their populations were much higher. The canola buffers of both experiments were colonized by *Myzus persicae* and *Lipaphis pseudobrassicae.* In both exps 2 and 3, the two types of symptoms that developed in plots with BYMV-infector plants were readily distinguishable. In both, within each entire plot, although the extent of initial symptomatic plant infections was the same for plants for necrotic BYMV symptoms in plots with necrotic strain infector plants as for non-necrotic BYMV symptoms in plots with non-necrotic strain infector plants (Figure 8G,H), subsequently, the non-necrotic strain spread faster, infecting more plants (Figure 8I,J). This resulted in the disease progress curve for the non-necrotic strain accelerating away subsequently (a polycyclic pattern), whereas that for the necrotic strain increased more slowly (a near-monocyclic pattern). The disease progress curves for the plot areas outside the 5 m diameter zones around the introduced infection foci differed from those in the entire plot in that the non-necrotic strain curve accelerated away even faster from the necrotic strain curve. The opposite transpired with the disease progress curves from within the 5 m diameter zones around the introduced infection foci, with the non-necrotic strain curve remaining closer to the necrotic strain curve. There was much less BYMV spread into the control plots, but the necrotic strain still infected more plants within them. In exp 3, there were no significant differences in BYMV incidences attributable to the time of focus removal, with the disease progress curves for each BYMV strain remaining very similar to each other regardless of whether infector plants were removed early or late. Also, although the final extent of the virus spread of each BYMV strain was more than double that which occurred with the same strain in the entire plots of exp 2, the overall findings in both experiments closely resembled each other, with those for the non-necrotic strain infections accelerating away from those with necrotic strain infections reaching much higher final incidences (Figure 9A,B) [77].

The two previous paragraphs in this section described studies on the temporal spread patterns that occurred when necrotic and non-necrotic BYMV strains infected narrow-leafed lupin growing in a natural spread situation or two field experiments (exps 2 and 3). In addition, these three investigations also provided detailed information about the spatial spread patterns of both BYMV strains [78]. Once the plants had died at the end of the growing period, the positions of all those marked individually with color-coded ribbons or stakes when they first developed symptoms were mapped. For this purpose, the selected mapping areas were sub-divided into square (1 × 1 m) quadrats. The areas selected were the natural spread site’s entire square (20 × 20 m) recording area, each of the three almost square (17 × 18 m) plots comprising one of exp 2’s replicates and each of the five rectangular (10 × 20 m) plots comprising one of exp 3’s replicates. The color codes identified the assessment date and whether the symptoms present were necrotic or non-necrotic. The SADIE program was used to quantify the spatial pattern revealed by these cumulative data. Briefly, for the non-necrotic strain at the natural spread site, there was a gradual increase in the amount of infected plant clustering over time, whereas, for the necrotic strain, not only was such clustering smaller, but also it failed to increase over time (Figure 9C). Within the plots of both of exps 2 and 3 with infector plants, although the clustering was greater with the non-necrotic strain, there was also a gradual increase in clustering with the necrotic strain. In exp 3, however, within its plot which lacked introduced infector plants, although there was no significant clustering for the necrotic strain, there was with the non-necrotic strain (Figure 9D). Moreover, in all instances, when the clustering data for successive assessment dates were tested for association, there was a positive association for both BYMV strains. However, although this association was stronger with the non-necrotic strain, it declined with the increasing time lag. In addition, as the period in between the initial infection and the appearance of symptoms following systemic spread was 2–3 weeks, this association was strongest between the assessments that were closest to each other. Contour maps for the local association between assessment dates found that the spatial associations from the coincidence of infection patches were weaker than those from the coincidence of infection gaps (Figure 9C,D) [78]. In summary, the spatial clustering and association analyses found that BYMV’s necrotic strain spread more slowly and less comprehensively than its non-necrotic strain. In all instances, more clustering occurred with the non-necrotic than the necrotic strain, whereas the necrotic strain spread resulted in more diffuse infection associated with more infection gaps.

The two field experiments (exps 2 and 3) described previously in this section also provided seed yield information [68]. For exp 2, tagged plants that developed BYMV necrotic or non-necrotic strain symptoms at seven different plant-growth stages were collected at random from plots with necrotic or non-necrotic strain infector plant foci, respectively (50 plants at each growth stage/BYMV strain). After oven drying, data were collected from each plant for the shoot dry weight, seed weight and seed number and these seeds were passed through different mesh-sized sieves. For exp 3, all seeds were harvested from each plot and their overall seed yields and seed weights based on 500 seeds/plot were determined. In addition, the seeds were passed through different mesh-sized sieves. In exp 2, at the two earliest assessment dates, necrotic BYMV infection killed infected plants before any seeds developed. The extents of the shoot dry weight/plant losses obtained were dependent upon when the symptoms first appeared: they were 27–88% (non-necrotic strain) and 55–98% (necrotic strain). The seed yield was reduced by 48–99% (non-necrotic strain) and 80–100% (necrotic strain) and the seed number by 35–98% (non-necrotic strain) and 74–100% (necrotic strain). The seed size was diminished by both strains, but this effect was far higher with the necrotic strain, especially with early infection. Thus, the reduced seed number and size both contributed to the seed yield losses. In exp 3, the symptomatic plant incidences at the final assessment within all plots with non-necrotic infection foci, necrotic infection foci or without any infection foci (=control plots) were 50%, 32% and 22%, respectively, and the seed yield of the plots with non-necrotic infection foci was 39% and 27% smaller than those of the control plots and plots with necrotic infection foci, respectively. The plots with non-necrotic infection foci produced significantly lower seed yields than those of the other two treatments. In contrast, there was no significant difference between the seed yields of plots with necrotic infection foci and those of the control plots and no significant difference due to the infection focus removal time. The 500 seed weights for plots with necrotic infection foci or without any infection foci were significantly larger than those for plots with non-necrotic infection foci. Also, the largest seed fraction sizes (5–5.5 and >5.5 mm) were significantly larger for plots with the necrotic strain than with the non-necrotic strain infection foci. How can the 39% difference in yield between plots with 22% (control plots) and 50% (plots with non-necrotic foci) symptomatic plant incidences be explained despite the only 28% final incidence difference between them? The incidences recorded at final assessment must have been underestimated, resulting from factors like (i) the delay between the systemic invasion of lupin plants and the appearance of readily visible non-necrotic symptoms, (ii) further BYMV spread having occurred before the cessation of crop growth which took place up to a month afterwards, especially in the plots with non-necrotic foci, and (iii) healthy plants growing over less vigorous, recently infected plants resulting in these being missed beneath the canopy.

In 2001, two additional field experiments at different WA sites (exps 4 and 5) studied the temporal patterns of BYMV non-necrotic strain spread and the resulting seed yield losses that occurred in narrow-leafed lupin stands [68]. A range of initial infection foci were introduced to their rectangular (5.6 × 15 m) plots to generate different infection incidences. The plots were sown with cv. Tanjil seed at seeding rates of 60 kg/ha (exp 4) or 80 kg/ha (exp 5) and row spacings of 35 cm (exp 4) or 22 cm (exp 5) and a 5 m wide non-host barrier of canola surrounded each of them (Figure 9E). BYMV-infected infector transplants of subterranean clover both infected with non-necrotic strain isolate LKoj1-NN and infested with *Myzus persicae* were introduced to act as virus sources for spread by aphids (Figure 9F,G). The four treatments were each replicated six times and consisted of plots without any infector plants or with pairs of infector plants transplanted at 4, 8 or 16 evenly spaced foci/plot. Leaf samples were collected at random on seven assessment dates and the aphid species present were recorded. The samples were tested for BYMV by ELISA, and the virus incidence data obtained for each treatment were plotted as pathogen progress curves. In addition, the aphid numbers on shoot growing tips were counted on three (exp 5) or four (exp 4) assessment dates. *Myzus persicae*, *A. craccivora* and *Acyrthosiphon kondoi* colonized the lupin plants in both experiments, but the aphid population was lower in exp 4 (maximum three/shoot tip) than exp 5 (maximum ten/shoot tip). In both experiments, the pathogen progress curves revealed that the numbers of infector plant foci/plot determined the extent of the non-necrotic strain spread that occurred, this being least in the plots with none, in which there was very little (exp 5) or none (exp 4), and greatest in the plots of the treatment with 16 infector plant foci. A statistical analysis of the Area Under the Pathogen Progress Curve (AUPPC) data showed that for each increase in foci number/treatment, there was a significant increase in virus spread. The spread pattern was always polycyclic. In exp 4, all seeds were harvested from each plot and their overall seed yields were determined. In exp 5, a 5.4 × 15 m strip in the middle of each plot was harvested. For each plot, the overall seed yields and seed weights based on 500 seeds/plot were determined. For exp 4, in the treatments with 4, 8 or 16 foci/plot, the seed yields recorded were 14%, 31% and 64% smaller than those of the control plots, respectively. A statistical analysis of the treatment seed yield data values found that, apart from those with four and eight foci/plot, all were significantly different from each other. In exp 5, the corresponding seed yield losses were 21%, 43% and 66% smaller for the treatments with 4, 8 or 16 foci/plot, and, in this instance, the statistical analysis of seed yield data found that all seed yield values/treatment were significantly different from each other. Their mean 500-seed weight values for the treatments with 0, 4, 8 or 16 foci/plot were 81.2, 79.2, 70.8 and 61.1 g. The values for treatments with 8 or 16 foci/plot were significantly different than each other and from those with 0 or 4 foci/plot. In contrast, the zero and four foci/plot treatment values were not significantly different from each other. As with exp 3 in the previous paragraph, the yield losses obtained with non-necrotic BYMV infection in exps 4 and 5 were greater than would be expected from the final % incidences recorded, e.g., in plots with 16 infection foci/plot, yield losses reached 64–66% when the corresponding final virus incidences recorded were only 23–27%. Similarly, in a further field experiment with narrow-leafed lupin in 2003 at the same site as exp 5 in 2002, a 50% non-necrotic strain incidence at the final assessment resulted in a 77% seed yield loss [91]. Although these incidence assessments were all based on sample testing rather recording plants with symptoms, as carried out in exp 3, the incidences recorded at the final assessment must still have been underestimated. The three factors likely to have caused this incidence underestimation are the same as those described at the end of the previous paragraph.

In conclusion, this research reveals two important aspects of strain-specific systemic hypersensitive resistance (in this case, involving gene *Nbm-1*) when it is deployed in the field. Firstly, that by killing plants, thereby removing them as sources of virus infection within a crop, where it can effectively limit the spread of the virus strains that elicit it (in this case, necrotic strain BYMV). Thus, unlike localized hypersensitive resistance which operates at the single plant level, systemic hypersensitive resistance operates at the plant population level. Secondly, because BYMV-infected narrow-leafed lupin plants infected with the non-necrotic strain remain alive instead of being killed, as occurs with the non-necrotic strain, the non-necrotic strain spreads faster, causing greater yield losses. This is because only plants infected with the non-necrotic strain remain to act as significant primary infection foci for further spread by aphid vectors. Thus, strain-specific systemic hypersensitive resistance becomes ineffective when a resistance breaking strain is spreading within a crop.

### 5.6. Epidemic Drivers and Forecasting

Section 4 described the Australian information currently available about the many alternative BYMV host species with the potential to act as external sources for the virus spread to lupin crops. Subterranean clover pastures are by far the most important of these. Section 4, Section 5.3 and Section 5.5 described the aphid vector species found associated with BYMV spread in field experiments with narrow-leafed lupin. For the five most important lupin-colonizing or non-lupin-colonizing aphid vector species in the WA grainbelt, Jones [90] summarized the aphid vector situation within the growing season as follows: “*Myzus persicae*, *A. craccivora* and *Acyrthrosiphon kondoi* colonize lupin crops, *Myzus persicae* and *L. pseudobrassicae* colonize adjacent canola crops and wild radish (*Raphanus raphanistrum*) weeds, and *R. padi* colonize adjacent cereals and grasses. In mixed species pasture dominated by subterranean clover, *Acyrthrosiphon kondoi* and *A. craccivora* colonize clovers and other legume species, *Myzus persicae* mainly colonize broad-leafed weeds and *R. padi* colonize grasses. All five species are involved to differing extents in virus transmission to and within lupin crops, the actual vector species scenario varying with site and year”.

**Figure 10:**
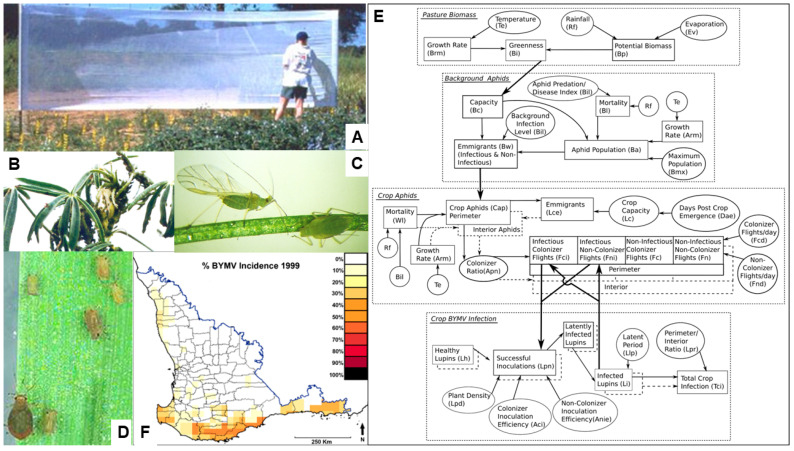
Images from aphid vector, epidemiology and forecasting studies with bean yellow mosaic virus (BYMV) in narrow-leafed lupin (*Lupinus angustifolius*). (**A**) Net employed to trap and collect flying airborne winged vector aphids downwind of virus-infected lupin stands for use in virus transmission studies (at South Perth in 1996). (**B**) Dense population of critical BYMV vector aphid *Myzus persicae* (green peach aphid) colonizing lupin shoot. (**C**) Example of alate and apterous BYMV vector aphid *Acyrthosiphon kondoi* (bluegreen aphid) colonizing a leaf petiole. (**D**) Example of apterous adult and nymphal growth stages of BYMV vector *Rhopalosiphum padi* (oat aphid) colonizing a cereal leaf. (**E**) Diagrammatic overview summarizing the four main components of the overall forecasting model for BYMV infection of lupin crops (pasture biomass, background aphids, crop aphids and crop BYMV infection) and how they are inter-related. Solid outlines represent parameters and variables for perimeter populations of aphid vectors and lupins or those that are equal for both perimeter and interior populations; dashed outlines represent variables from the interior populations of aphid vectors and lupins. (**F**) BYMV-lupin pathosystem risk map produced for 1999 by the modeling framework for different districts in the southwest Australian grainbelt. Colors represent 90–100% to 0–10% BYMV incidence in lupin crops. (**C**) Image credit @Department of Primary Industries and Rural Development/Deborah Thackray.

In 1993–1996, the roles of different aphid species as lupin colonizing and non-colonizing BYMV vectors were investigated [18]. When acquisition access times of 5–10 min were used, the BYMV transmission efficiencies from infected subterranean clover to narrow-leafed lupin found were 77%, 15% and 14% for *Myzus persicae*, *Acyrthosiphon kondoi* and *A. craccivora*, respectively. Also, with 5–10 min acquisition access times, the BYMV transmission efficiencies from subterranean clover to lupin of six additional non-lupin-colonizing aphid species were *L. pseudobrassicae* 8%, *R. maidis* 6%, *R. padi* 5%, *Therioaphis trifolii* 5%, *Sitobion miscanthi* 11% and *B. rumexicolens* 0%. Over a 3-year period, vertically oriented nets (Figure 10A) were placed to catch flying aphids downwind of lupin BYMV resistance screening trials at an irrigated site in WA (Figure 7A,B). Each aphid was removed from the net individually, transferred in situ to a narrow-leafed lupin plant (one aphid/plant), left to probe for 1 h and then preserved for subsequent species identification. The numbers of aphids that transmitted BYMV were 11/727 (=2%) and the aphid species that transmitted it were the same ones as the three most abundant species caught: *Acyrthosiphon kondoi*, *Myzus persicae* and *R. padi,* which constituted 16%, 9% and 39% of the total catch, respectively (Figure 10B–D). This study suggested that the lupin-colonizing species *Myzus persicae* and *Acyrthosiphon kondoi* and non-lupin-colonizing species *R. padi* are the most important BYMV vectors in lupin crops in WA. However, *A. craccivora*, *L. pseudobrassicae*, *R. maidis*, *R. padi*, *T. trifolii* and *Sitobion miscanthi* may sometimes play significant roles depending upon their local abundance.

In 1998 and 1999, ‘data collection’ blocks of narrow-leafed lupin representing different rainfall and geographical regions of the WA grainbelt were sown with 7% CMV-infected cv. Gungurru seed at the same four sites each year. These were dual-purpose blocks used to collect data on both CMV and BYMV spread. Each block was located next to a subterranean clover pasture, which acted as an external BYMV infection source for its spread into the lupin block [20,92,93,94]. Climatic data on daily fluctuations in temperature, rainfall and wind strength and direction were obtained from the nearest weather station. The biological data collected from each block included (i) lupin plant density; (ii) date of first aphid vector arrival; (iii) increase in BYMV incidence in lupin plants throughout the growing season, enabling BYMV infection progress curves to be plotted; (iv) numbers of each aphid species colonizing the lupin plants; and (v) numbers of each colonizing and non-colonizing aphid species trapped flying overhead. This information, similar data collected from field experiments carried out over a 14-year period at widely dispersed WA grainbelt sites, combined with data from glasshouse experiments (Section 4, Section 5.3 and Section 5.5 above) provided a sound understanding of the factors driving the range of scenarios that unfolded at different sites in different years [16,20,92,93,94,95]. In brief, in narrow-leafed lupin crops, the magnitude of the BYMV epidemics and seed yield losses that arise differ widely with the year, growing season, rainfall zone and geographical region. The principal drivers that determine what transpires are (i) the size of the primary external virus infection source (i.e., BYMV-infected subterranean clover pasture) and how close it is to the crop; (ii) the growth stage the crop has reached when aphid vectors first arrive; (iii) the magnitude of the aphid population and extent of their activity; (iv) the species of aphids that visit the crop and whether they colonize it; (v) weather parameters, especially, the temperature, rainfall and evaporation; and (vi) cultural practices that influence the amount of groundcover, plant density and stage of crop growth when canopy closure occurs [16,20,53,64,90,94]. BYMV spread by aphid vectors was increased by sowing at low seeding rates with wide row spacing as this delayed canopy closure and lack of ground cover which encouraged aphid landings. In narrow-leafed lupin crops, factors such as the rainfall, wind and temperature influence the extent of virus transmission, aphid numbers and aphid behavior. Heavy rainfall and high winds knock aphids off plants, reducing the aphid population and resulting virus transmission, but moist, warm conditions enable plants to flourish, favouring aphid population growth and consequent virus transmission. Aphids are unable to reproduce sexually under Mediterranean-type climate conditions where rainfall is minimal in summer and early autumn. Over the hot, dry summer period, they persist in very low numbers upon herbaceous weed or volunteer crop host plants growing in scarce, damp locations which occur throughout the WA grainbelt. These damp spots include roadside ditches where dew runoff from roads and tracks provides sufficient moisture for plants to survive in, soaks where moisture reaches the surface, creek edges and irrigated gardens. The timing and amount of rainfall in the first two months of autumn (March and April) determines the extent of the aphid build-up before lupin crops are sown in late autumn (May) and when they first arrive in growing lupin crops. The consequence of having substantial early rain is that it allows a ‘green ramp’ of annual pasture, weed and volunteer crop hosts of aphids to emerge and flourish. While building up their populations on plants within subterranean clover pastures, aphid vectors acquire BYMV from seed-infected clover plants and spread BYMV within these pastures. Subsequently, their early flights to emerging lupin crops then introduce BYMV, giving rise to its early spread, prolonged aphid vector activity and widespread BYMV infection, which, in turn, results in greater seed yield losses. In contrast, when the rainfall before sowing time is light and late, the opposite outcome of minimal BYMV spread and seed yield losses occurs in lupin crops [16,20,94,95].

Based on the knowledge of epidemic drivers summarized above, a hybrid mechanistic/statistical model was developed that forecasts aphid vector activity and BYMV epidemics in narrow-leafed lupin crops in the WA grainbelt [20,94,95]. Its mechanistic approach enabled it to model individual pathosystem components with greater flexibility (Figure 10E). It used daily rainfall and mean temperature during late summer and early autumn to predict aphid population increase, aphid flights from self-regenerating subterranean clover pasture to lupin crops and BYMV spread. Four major processes were included in its calculations: (i) clover pasture biomass accumulation; (ii) pasture aphid population increase and activity; (iii) infectious aphid migration to the lupin crop perimeter and from there into the crop; and (iv) BYMV’s within-crop multiplication, movement and spread by aphids. The model predicted aphid vector arrival time and necrotic strain BYMV spread successfully in seven of the eight datasets provided by the 1998 and 1999 lupin ‘data collection’ blocks (Figure 10F). In the one instance where its prediction was poor, which was in 1998 at the southernmost site, the biomass incidence remained low for a month longer than in 1999 at the same site. This delayed the aphid population increase in pastures, resulting in a later time of aphid first arrival and, ultimately, a lower final BYMV incidence in the lupin block. Two factors not accounted for in the model were likely reasons for this delay in pasture biomass development. They were frost and extreme wind conditions. Otherwise, there was always correspondence between predicted and actual aphid numbers and BYMV incidence values in the other seven datasets. A sensitivity analysis of factors likely to alter the outcome found that the model responded credibly across the likely range of pasture BYMV incidences, lupin emergence dates and plant densities. This suggested that BYMV control measures focusing on crop hygiene and rapid canopy closure are likely to be effective as part of an IDM strategy. The model’s accuracy was adequate for its use as a guide to what BYMV management practices farmers should deploy at sowing time. Therefore, an automated weather data collection system was added to the model so that it could provide a prompt BYMV forecast for lupin crops growing in different regions of the WA grainbelt.

### 5.7. Integrated Disease Management

In Australia, by the early 2000s, the BYMV narrow-leafed lupin pathosystem was well understood, and the IDM strategy devised to address this major lupin disease epidemic had been adopted widely in WA [16,85,96,97]. The strategy developed for commercial lupin crops was based on the results of the 14-year research program described above. It consisted of phytosanitary, cultural, chemical and host resistance control measures which act in different ways and target either the primary virus source (internal or external) or virus spread (early or later phases) [97,98]. It was devised to manage both necrotic and non-necrotic BYMV strains. It’s most important components were sowing a non-host perimeter strip in between adjacent pasture and the lupin crop, maximizing the stubble groundcover within the lupin crop and promoting rapid canopy closure by sowing lupins at high seeding rates with narrow row spacing. Its diverse components were as follows:

- Sow a perimeter non-host barrier crop between the adjacent subterranean clover pasture and the lupin crop to provide a ‘virus cleansing’ barrier that diminishes BYMV spread from external sources into the crop (cultural).
- Avoid fields with a large perimeter: area ratios adjacent to pastures to diminish BYMV spread from external sources into the crop (phytosanitary).
- Sow seeds at high seeding rates to generate high plant densities and early canopy closure to (i) shade out early–current season infected plants, thereby minimizing the early internal secondary infection source for subsequent BYMV spread by aphid vectors, and (ii) diminish aphid landing rates, thereby further diminishing BYMV spread (cultural).
- Sow seeds at narrow row spacing to generate early canopy cover, thereby diminishing aphid landing rates and the extent of BYMV spread before canopy closure (cultural).
- Sow early maturing cultivars to diminish late BYMV spread by vector aphids, especially in prolonged growing seasons (cultural).
- Maximize stubble groundcover using minimum tillage procedures that minimize soil cultivation to diminish aphid landing rates, thereby reducing BYMV spread before canopy closure (cultural).
- Wherever possible, ensure isolation from neighboring subterranean clover pastures to avoid ingress of BYMV from vector aphids flying from external pasture sources (phytosanitary).
- Ensure isolation from neighboring pulse (including lupin) crops to avoid any ingress of BYMV from vector aphids flying from external crop sources (phytosanitary).
- Maximize weed control using selective herbicide to minimize potential clover or other weed infection sources of BYMV within the lupin crop (chemical).
- Spray adjacent pasture with pyrethroid insecticide to suppress both aphid vectors directly and BYMV spread indirectly within the main BYMV source for spread to lupins (chemical)*.

An extra item for special-purpose lupin crops is as follows:

- Mixed cropping with a non-host (e.g., cereal) to diminish BYMV spread to lupins grown for hay production (cultural).

* Note: this recommendation is derived from studies in grazed annual pasture swards where a non-persistently aphid transmitted virus was spread by apterous *Acyrthosiphon kondoi* and this spread was controlled effectively by pyrethroid insecticide application [99].

### 5.8. Main Research Achievements

Australian research on the BYMV/lupin pathosystem demonstrated the following:

- The foliage symptom types and extent of yield losses that BYMV infection causes in different lupin crop species.
- Subterranean clover pasture is the most important external source for BYMV spread to lupin crops, although other alternative BYMV host species can sometimes also act in this way.
- The lupin-colonizing species *Myzus persicae* and *Acyrthosiphon kondoi* and non-lupin-colonizing species *R. padi* are the most important BYMV vectors in lupin crops, but *A. craccivora*, *L. pseudobrassicae*, *R. maidis*, *R. padi*, *T. trifolii* and *Sitobion miscanthi* can sometimes also paly significant roles as its vectors.
- The widespread occurrence of necrotic and non-necrotic BYMV strains in narrow-leafed lupin, the seed yield losses they cause and their lack of seed-to-seedling transmission despite BYMV being seed-borne in yellow and white lupins.
- Late infection (i.e., after first flowering) with BYMV necrotic strain causes BPS in narrow-leafed lupin.
- Reflective mulch reduces BYMV spread to narrow-leafed lupin breeder’s single-row plots.
- Non-host borders around crop perimeters provide a ‘virus-cleansing’ barrier which diminishes the BYMV spread from external virus sources into lupin crops. Similarly, growing a non-host crop in a mixture with lupin decreases the BYMV spread to the lupin plants.
- Cereal straw spread on the soil surface to simulate stubble retention and sowing the seed at narrow row spacing using high seeding rates to achieve early canopy closure both diminish aphid vector landings and the consequent BYMV spread, with the straw acting before canopy closure takes over.
- Two independently inherited strain-specific BYMV hypersensitivity genes occur in narrow-leafed lupin, one controlled by a single dominant hypersensitivity gene, *Nbm-1,* and the other by an, as yet uncharacterized, hypersensitivity gene. Differential genotypes with or without these genes distinguish four biological strain groups (=pathotypes) of BYMV.
- Strain-specific systemic hypersensitive resistance controlled by the gene *Nbm-1* operates at the plant population level, limiting BYMV necrotic strain spread by killing infected plants, which removes them as internal virus infection sources for its spread by aphid vectors. This resistance is ineffective when the non-necrotic (i.e., resistance breaking) BYMV strain is present as the infected plants remain alive, providing infection foci for further spread, resulting in greater overall yield losses.
- The delayed BPS development trait in cv. Jenabillup could be important in future breeding for resistance to BPS in narrow-leafed lupin, so knowledge of its likely nature (resistance to systemic infection via the phloem or mature plant resistance, etc.) and its genetic basis (likely to be polygenic) warrant further investigation.
- Narrow-leafed lupin breeding line 84A086-5-20-31 has a high level of BYMV ‘infection resistance’. Therefore, it is recommended for use as a parent in crosses aimed at producing new lupin cultivars suitable for BYMV-prone high-rainfall regions.
- Attempts to introduce BYMV resistance to narrow-leafed and yellow lupin using viral protease (NIa) and replicase (NIb) genes proved unsuccessful despite initially encouraging findings.
- Diverse factors influence the temporal and spatial dynamics of BYMV spread in narrow-leafed lupin stands in different ways depending on the circumstances. These include whether its spread is from external or internal virus sources, necrotic or non-necrotic strains (or both) are present, first aphid vector arrival is early or late, straw is present or absent on the soil surface, row spacing is narrow or wide, seeding rates are low or high and non-host borders are present or absent.
- BYMV epidemics and the resulting seed yield losses in narrow-leafed lupin crops differ widely with the year, growing season, rainfall zone and geographical region. The main epidemic drivers are (i) the size of the primary external virus source and its proximity; (ii) the crop growth stage when aphid vectors first arrive, the size of the aphid population and the extent of their activity; (iii) the aphid species that visit the crop and whether they colonize it; (iv) weather parameters, especially the temperature, rainfall and evaporation; and (v) cultural practices which influence the amount of groundcover, plant density and stage of crop growth when canopy closure first develops.
- A hybrid mechanistic/statistical model was developed which forecasts aphid vector activity and BYMV epidemics in narrow-leafed lupin crops. It uses daily rainfall and the mean temperature in late summer and early autumn to predict aphid population increase, flights from clover pasture and BYMV spread.
- The IDM strategy devised for lupin crops includes phytosanitary, cultural, chemical and host resistance control measures that act in different ways and target either the primary BYMV source (internal or external) or virus spread (early or later phases). Its main components were sowing a non-host perimeter strip in between adjacent pasture and the lupin crop, maximizing the stubble groundcover within the crop and promoting rapid canopy closure by sowing at high seeding rates with narrow row spacing.

### 5.9. Further Research

The existing BYMV/narrow-leafed lupin IDM strategy consisting predominantly of cultural and phytosanitary measures should remain in use, especially in the more BYMV-prone high-rainfall grainbelt zones. However, although it has proven effective against early infection with the necrotic BYMV strain, additional research is required to better understand and manage BPS resulting from late BYMV infection (Section 5.2 above). Also, further field experimentation to make improvements to cultural control measures, such as optimizing the stubble retention recommendation to improve aphid vector landing deterrence (Section 5.3 above), would help increase its overall effectiveness. Moreover, as the non-necrotic BYMV strain breaks the hypersensitive resistance gene, *Nbm-1,* present in all Australian narrow-leafed lupin cultivars and this breakdown results in greater infected plant numbers and consequent yield losses (Section 5.5 above), research is also required to better comprehend and manage it. In addition, given the likely importance of sowing BYMV-infected seed stocks in providing internal primary virus infection sources (i.e., seed-infected plants), the seed-borne BYMV strain found recently in white lupin in NSW (Section 5.1) needs attention. This is because the effective management of readily seed-borne viruses in cool-season pulse cropping requires using seed stocks that only have low levels of virus contamination when commercial crops are sown [100,101]. It also depends on pulse breeding programs distributing healthy seed stocks when they release new cultivars. Although field experiment studies to establish ‘threshold’ values for percentage seed infection that offer acceptable levels of risk of economic losses resulting from sowing virus-infected seed stocks in different grainbelt regions have already been established in Australia for CMV-infected narrow-leafed lupin seed [5,101,102] and PSbMV-infected field pea seed [103], none have been determined as yet for sowing the BYMV-infected seed of any cool-season pulses. Such field studies are now required with BYMV in white lupin, especially in NSW.

Further research is also required on host resistance to BYMV in lupins. The studies summarized above (Section 5.2) did not find the cause of the delayed BPS symptom expression present in cultivars like cv. Jenabillup, but not in cultivars such as Mandelup which express BPS more rapidly, resulting in greater seed yield losses. Since this delayed symptom expression trait could be important when breeding new BPS-resistant lupin cultivars, obtaining an understanding of its likely cause (resistance to systemic infection via the phloem, mature plant resistance, etc.) and genetic basis (likely to be polygenic) warrants further investigation. There is also a need to identify the suspected additional BYMV-hypersensitive resistance gene in breeding line 90L423-07-13 and accession no. P26697 (Section 5.4 above) so it can be used in breeding new narrow-leafed lupin cultivars. Furthermore, research is also necessary to understand the nature of the BYMV ‘infection resistance’ trait found in narrow-leafed lupin breeding line 84A086-5-20-31 (Section 5.4 above). This trait depends upon requiring many more infectious vector aphids to establish BYMV infection successfully and is likely to be quantitatively inherited. Once understood, it could also be made available for use in future lupin cultivar breeding for BYMV resistance.

A review on host resistance to viruses in lupins published in 2023 [75] made other suggestions for future research. In brief, those most relevant to lupin BYMV resistance breeding included (i) applying genetic modification approaches, especially CRISPR and RNAi, to obtain stable virus resistance [87,88,89,90]; (ii) streamlining the lupin breeding process by identifying quantitative trait loci (QTLs) to employ as molecular markers for the incorporation of polygenically inherited virus resistance traits [104]; and (iii) using speed breeding to accelerate the virus resistance breeding process [105].

A comprehensive understanding was assembled of the epidemic drivers that operate when BYMV infects narrow-leafed lupin crops in south-west WA lupin crops. This enabled the development of a forecasting model and DSS which predicted aphid vector arrival time and necrotic BYMV strain spread successfully. However, this forecasting model and DSS need to be updated to take account of recent alterations in the WA grainbelt climate (especially reduced rainfall and increased temperatures) and in the cultural practices used with lupin production. Its validation would require the establishment of further annual lupin ‘data collection’ blocks located at representative sites like those used in 1998 and 1999 (Section 5.6 above). Also, its adjustment and validation for use in eastern Australian grainbelts would require the establishment of similar ‘data collection’ blocks in other states.

## 6. Pulses Other than Lupins

**Figure 11:**
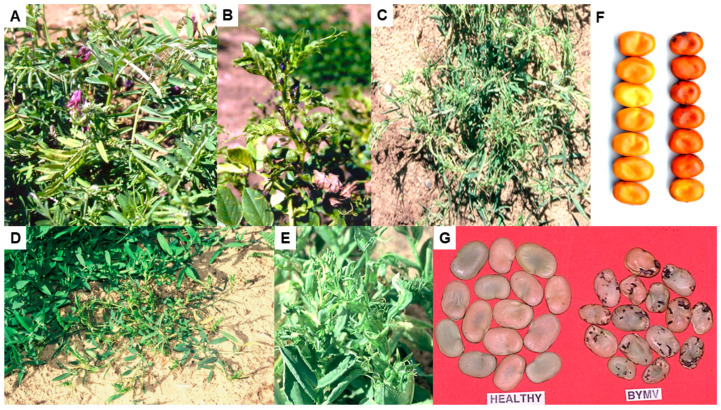
Images showing symptoms caused by bean yellow mosaic virus (BYMV) infection in foliage of minor cool-season pulses (**A**–**E**) and seeds of faba bean *(Vicia faba*) (**F**,**G**). (**A**) Plant of common vetch (*Vicia sativa*) with symptoms of yellow mosaic in leaflets of young leaves infected with BYMV (at South Perth in 1998). (**B**) Plant of narbon bean (*V. narbonensis*) with severe foliage symptoms consisting of leaf chlorotic mosaic, deformation and reduced size and plant stunting (at South Perth in 1998). (**C**) Plants of grass pea (*Lathyrus sativus*) with severe symptoms consisting of leaf chlorosis, deformation and reduced size, and plant stunting (at South Perth in 1998). (**D**) Plant of dwarf chickling (*L. cicera*) with severe symptoms consisting of leaf chlorotic mosaic, deformation and reduced size and plant stunting (center) and healthy plant within same row (upper left) (at South Perth in 1997). (**E**) Plant of *L. ochrus* with severe foliage symptoms in young leaves consisting of leaf palor, deformation and reduced size (at South Perth in 1997). (**F**) Row of faba bean cv. Fiesta seeds from BYMV-infected plant with surface necrotic markings, malformation and size reduction (right) and row of seeds from healthy plant (left) (at Avondale in 1999). (**G**) Seeds harvested from BYMV-infected faba bean plants growing in the Middle East with surface necrotic markings, malformation and size reduction (right) or from healthy plants (left). (**G**) Image credit@International Center for Agricultural Research in the Dry Areas/Khaled Makkouk.

As explained in Section 2 and Section 3 above, since BYMV was first reported to be present in 1943, a considerable amount of BYMV research has been completed around Australia. Initially, Australian BYMV research on cool-season pulse crops other than lupin was undertaken in NSW, QLD, TAS and WA and focused mainly on infection of field pea and faba bean [2]. Subsequently, it expanded to include the major pulses, lentil and chickpea, and the minor pulses, such as bitter vetch, narbon bean, grass pea, dwarf chicking, *L. clymenum* and *L. ochrus* [24,26]. In faba bean, BYMV symptoms ranged from leaf-vein clearing, mosaic and deformation with mild plant stunting to asymptomatic infection (Figure 2E). In field pea, they varied from leaf mosaic and deformation, severe plant stunting and premature senescence to symptomless infection (Figure 2F). In lentil, they varied from leaf-vein clearing, severe mosaic, chlorosis and deformation, severe plant stunting and premature senescence to leaf-vein clearing and mild leaf mosaic with mild plant stunting (Figure 2D). In chickpea, they were leaf chlorosis (kabuli) or reddening (desi), apical necrosis, plant stunting and premature senescence (Figure 2G). In common vetch, they ranged from leaf severe mosaic, chlorosis and leaf deformation with plant stunting to mild leaf mosaic (Figure 11A). In narbon bean, they were leaf-vein clearing, severe mosaic, chlorosis and deformation with necrotic stem streaking, apical necrosis, severe plant stunting and premature senescence (Figure 11B). In grass pea, they ranged from leaf-vein clearing, followed by severe leaf mosaic and deformation and severe plant stunting to leaf-vein clearing and mild mosaic and deformation with mild plant stunting (Figure 11C). In dwarf chickling (Figure 11D), *L. ochrus* (Figure 11E) and *L. clymenum,* individual leaf and overall plant foliage symptom severity were both high, consisting of obvious leaf-vein clearing, mosaic, chlorosis and deformation and plant stunting with or without premature senescence [25]. Sometimes, in addition to decreasing seed size, BYMV infection also damages seed quality by causing malformation and discoloration. For example, irregular necrotic spots and discoloration sometimes develop on the seedcoats of seeds from infected faba bean plants (Figure 11F,G) [106,107,108].

### 6.1. Occurrence in Plots, Crops and Seed Stocks

In 1986–1987, BYMV was detected in 25/40 leaf samples from faba bean plots growing in TAS [17]. More detailed information concerning BYMV’s occurrence in cool-season pulses growing in different Australian states came later from surveys of experimental plots and field crops as well as seed stocks. In 1994–1995, no BYMV was detected in a survey of commercial crops of chickpea (63) and lentil (28) growing in agricultural areas of south-west WA [109]. Similarly, no BYMV was detected in surveys of crops and field experiments of chickpea (12) and lentil (9) growing at experimental sites in the same region. However, BYMV was detected in plots of different genotypes of chickpea and lentil growing at the South Perth site. Lentil plots were frequently BYMV-infected in both years, e.g., in 1995, 23/35 plots were infected. In contrast, with chickpea, only three plots became infected in 1994 and none in 1995. This suggested high susceptibility in the lentil genotypes, but low susceptibility in the chickpea genotypes. Tests for BYMV seed-to-seedling transmission in lentil found 0.8% with seed from a mixture of different genotypes and 3% with seed of cv. Laird [109]. In 1994–1999, surveys of experimental plots in south-west WA, found BYMV infecting symptomatic plants of field pea and faba bean. Plots of the minor pulses, narbon bean, grass pea, dwarf chickling, bitter vetch and *L. clymenum,* were similarly affected [26]. In 1998–1999, larger-scale surveys of commercial faba bean and field pea crops growing in the same region found the incidence of BYMV-infected crops within any one year was up to 35% in faba bean and 18% in field pea, with the within-crop incidence reaching 31% in faba bean and 11% in field pea. A dwarf chickling crop had 4% BYMV infection [26]. In 1998 and 1999, tests on commercial seed stocks found BYMV in 1/11 faba bean and 3/30 field pea seed samples; BYMV incidences in seedlings from these infected samples were relatively low: 0.4% (faba bean) and 0.1–0.8% (field pea) [26]. In 2000–2005, annual surveys of lentil, faba bean and chickpea crops in VIC and SA sometimes detected BYMV, but were only published in a brief conference abstract format so details of the extent of crop infection are no longer accessible [110,111,112,113]. In 2006, a survey of field pea crops in southern NSW and VIC and faba bean crops in NSW detected BYMV in (i) 1/21 and 1/10 field pea crops from NSW and VIC, respectively, at an incidence of 1–3% in the two infected crops found and (ii) 1/3 faba bean crops from NSW at an incidence of 1% [114]. In 2012, 12 chickpea crops growing in the Liverpool Plains in NSW were surveyed by collecting and testing 240 symptomatic and 159 asymptomatic leaf samples. No BYMV was found. In contrast, BYMV was detected in 22/52 (i.e., 42%) of random leaf samples from a faba bean crop [115]. In 2020, during a major BYMV epidemic in faba bean crops in north-west NSW, infections occurred very early and resulted in severe foliage symptoms including leaf mosaic, deformation and necrosis and plant stunting, with some plant death. When 359 randomly collected faba bean leaf samples from several sites were tested, BYMV was detected in 44% of them, while amongst 234 leaf samples from plants with leaf mosaic symptoms, 89% of these were BYMV-infected [116].

### 6.2. Seed Yield Losses and Patterns of Spread

With the exception of the many yield loss studies described above with lupins (Section 5.1, Section 5.2, Section 5.3 and Section 5.5 above), only five Australian studies reported seed yield losses caused by BYMV in other cool-season pulses. Field studies in 1986–1988 in TAS found that BYMV diminished faba bean seed yield by reducing both overall pod numbers and numbers of seeds within them. Early BYMV infection resulted in greater losses than late infection (% yield losses not reported) [17]. During 1994–1998 in WA, in glasshouse tests, yield losses of 100% occurred with BYMV-infected dwarf chickling and narbon bean plants and very little or no seed formed on BYMV-infected plants of *L. ochrus*. Also, in BYMV genotype screening experiments in the field plants of the dwarf chickling and narbon bean genotypes tested developed very severe symptoms, failing to produce any seed with early-growth-stage infections. In addition, the severe BYMV symptoms that developed in most infected lentil genotypes and the infected *L. ochrus* genotypes resulted in ‘very poor’ seed production [25]. In 2004 in NSW, within a field experiment with faba bean cvs Fiord, Fiesta and Cairo and breeding lines Ac1604 and Ac0674, early BYMV infection always caused seed discoloration and considerable seed yield losses (% yield losses not reported), except in the BYMV-resistant line, Ac1604. These yield losses were due to reduced pod formation rather than smaller pod size [107]. In 2020, field experiments with faba bean compared the seed yields from plots inoculated with BYMV mechanically with those from non-inoculated plots and found that early infections could have a major impact on their yield, with an over 50% yield reduction in cvs PBA Warda and PBA Nasma or even an up to 70% yield reduction in cv Fiesta [116]. In other continents, studies reporting seed yield loss data for BYMV include up to 96% losses in infected lentil and faba bean [117,118] and up to 62% losses in field pea [119].

In 1979–1980 in SA, spatial patterns of BYMV spread were studied in replicated faba bean field experiments [23]. The plots were sown in autumn and matured in spring. Faba bean plants in plot centers were inoculated with BYMV in early winter (June) in 1979 or late winter (August) in 1980. In both years, in half of these plots, 50 *A. craccivora* alatae were added to each inoculated infector plant and left to colonize them, but none were added to any of the infector plants in the other half of the plots. BYMV spread rapidly in both instances, causing the clumping of newly infected plants first around the original infector plants and then around secondary infection foci that developed as migrant aphid vectors which spread the virus further away. Secondary BYMV infection foci also appeared in the control plots without BYMV infector plants and, although the clumping of newly infected plants also developed around them, there was less spread overall. In both years, there was a minor aphid vector flight in autumn, but a major one in spring. The aphid species involved were *Myzus persicae*, *A. craccivora*, *Macrosiphum euphorbiae* and *Aulacorthum solani*. However, the relative importance of each species as BYMV vectors could not be established because their patterns of migration were similar [120]. In TAS in 1986–1988, additional field experiments also studied the patterns of BYMV spread in plots of faba bean [17]. BYMV infection foci were introduced at the plot centers and their spread was examined in relation to time of sowing, plant density, aphid flights and aphid infestation of faba bean plants. BYMV spread always radiated outwards from the initial infection foci. Most of the spread occurred in plots sown in early spring (September), whereas it was least in plots sown in late autumn (April or May). Faba bean plant density within plots and the types of neighboring crop species present were both important factors determining what transpired in the different years. In 1986–1987, *R. padi* was its principal aphid vector, but in 1989, the principal vector was *Acyrthosiphon pisum* [17].

### 6.3. Chemical and Cultural Control

In 1981, a field experiment in SA studied the effects of three insecticides and non-host barrier rows upon the extent of BYMV spread in replicated faba bean plots [120]. The insecticides used were the granular systemic organophosphate disulphoton applied to the soil at sowing time, the foliar systemic organothiophosphate demeton-S-methyl applied five times and the foliar contact organophosphate malathion applied eight times. An infector row of faba bean plants made up the central row of each plot. These infector plants were inoculated in situ by putting pieces of BYMV-infected faba bean stems infested with the aphid vector species *A. craccivora* and *A. pisum* (50 aphids of each species/plant) upon each of them. Two weeks beforehand, non-host barrier rows of barley, to which no insecticide was applied, were sown in single rows on either side (25 m away) of where each central BYMV infector row was to be sown. The systemic insecticides diminished aphid colonization of the plots, but none of the insecticide treatments decreased the BYMV spread. Conversely, the barley barrier rows had no effect on the aphid populations in the faba bean plants, but reduced the BYMV spread to the two rows nearest to them, although not to those further away. The BYMV spread was mainly by *Myzus persicae* and *Macrosiphum europhorbiae* migrants rather than the two species introduced to the infector plants. In TAS in 1986–1988, field experiments studied the effect of foliar applications of demeton-S-methyl and the foliar contact pyrethroid deltamethrin upon BYMV spread in faba bean plots [17]. No significant reduction in BYMV spread was obtained except in late autumn when infection rates in plots were low (further details unavailable). These field experiments suggest that, although applying insecticides to control BYMV spread is unlikely to be effective, non-host borders (e.g., of cereals) should be deployed to suppress BYMV spread by acting as ‘virus cleansing’ barriers (Section 5.3 above).

### 6.4. Host Resistance

**Figure 12:**
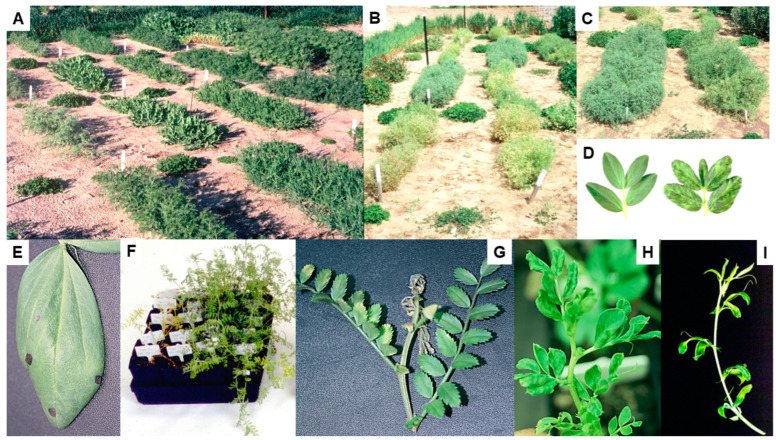
Images showing foliage symptoms caused by bean yellow mosaic virus (BYMV) infection from host resistance studies with cool-season pulses other than lupin. (**A**) BYMV resistance screening employing single-row plots containing cultivars, breeding lines and germplasm accessions of different cool-season pulses excluding lupin (*Lupinus* spp.). Subterranean clover (*Trifolium subterraneum*) transplants infected with BYMV necrotic strain isolate MI placed at both ends of each plot to ensure a uniform inoculum source for spread by naturally occurring aphids to the test rows (at South Perth in 1994). (**B**) BYMV resistance screening employing single-row plots containing lentil (*Lens culinaris*) cultivars, breeding lines and germplasm accessions; two healthy breeding line ILL7 136 rows visible and all other rows infected, showing leaf chlorosis, size reduction and premature senescence (at South Perth in 1997). (**C**) Single row of BYMV-resistant lentil breeding line ILL7 136 plants with healthy appearance (left) compared with susceptible plant row showing BYMV symptoms consisting of leaf chlorosis and plant stunting (right) (at South Perth in 1997). (**D**) Leaf of faba bean cv. Barkool (*Vicia faba*) plant sap inoculated with BYMV isolate LP showing severe mosaic symptoms (right) and uninocuated healthy leaf (left) (at South Perth in 1998). (**E**) Leaf of faba bean cv. Icarus sap inoculated with BYMV isolate MI causing necrotic local lesions without further virus spread, representative of localized hypersensitive resistance (at South Perth in 1998). (**F**) Lentil seedlings aphid inoculated with BYMV isolate MI showing symptoms of leaf pallor and premature senescence and severe plant stunting in susceptible cv. Digger (left) compared with healthy growth in breeding line ILL 7163 (right) (at South Perth in 1998). (**G**) Upper portion of chickpea (*Cicer arietinum*) cv. Heera shoot showing symptoms of apical necrosis from a plant that was aphid-inoculated with BYMV isolate LEsp-NN (at South Perth in 1998). (**H**) Upper portion of narbon bean (*V. narbonensis*) shoot showing leaf symptoms of severe mosaic, deformation and size reduction from a plant sap inoculated with BYMV isolate MI (at South Perth in 1998). (**I**) Upper portion of dwarf chickling (*Lathyrus cicera*) breeding line Lath-BC shoot showing leaf symptoms of severe mosaic, pallor, downcurling and size reduction from a plant aphid inoculated with BYMV isolate LWh-NN (at South Perth in 1998). (**A**,**B**) image credit@Department of Primary Industries and Rural Development/Simon McKirdy. (**E**,**G**,**H**) image credit@Department of Primary Industries and Rural Development/Yvonne Cheng.

In 1994–1998 in WA, the legume genotype BYMV field screening procedure of McKirdy and Jones [24] was used (Section 4 above). Seven field experiments studied the susceptibilities and sensitivities of pulses other than lupins to infection with BYMV isolate MI [25]. In each experiment, single row plots of each genotype were sown. In experiments 1–4, the plots of some genotypes were unreplicated, but all were replicated in experiments 5–7. In experiments 1 and 3–7, each row was exposed evenly to primary BYMV inoculum sources. This was carried out by transplanting the same numbers of infector plants, consisting of BYMV-infected subterranean clover plants in uniform arrangements (e.g., one at each plot end) (Figure 12A). In experiment 2, the aphid vectors spread it from nearby BYMV lupin-resistance screening experiments (Section 5.4 above). In all instances, BYMV was spread from the infection source plants to the test rows by naturally occurring aphids, especially *Myzus persicae*, *Acyrthosiphon kondoi* and *R. padi*. Overall, the pulses that proved most susceptible and sensitive (=vulnerable) to BYMV isolate MI infection were all genotypes of narbon bean, dwarf chickling and *L. ochrus* and most genotypes of lentil (Figure 12B). In contrast, field pea (three cultivars), faba bean (four cultivars), chickpea (eight cultivars), common vetch and grass pea were all ranked as having resistance. The BYMV-MI infection incidences in the field pea and faba bean cultivars ranked as resistant were 1–5%. In contrast, in chickpea they were only <1% (two cultivars) or 0% (six cultivars), which places the latter in the highly resistant category. Amongst the species with resistance rankings, chickpea was always highly sensitive, but the sensitivity rankings allocated to field pea, faba bean, chickpea, common vetch and grass pea varied depending upon the genotype present. Lentil was the most seriously affected major pulse species, e.g., 58/68 lentil genotypes were ranked as highly susceptible in experiment 7. Amongst the other ten lentil genotypes in experiment 7, one (ILL7163) was highly resistant (Figure 12B,C) while the others were ranked as resistant (two), moderately resistant (four), or susceptible (three). Following glasshouse inoculations with different BYMV isolates, lentil ILL7163 proved to be of particular interest, as it apparently possessed extreme BYMV resistance likely to be of use to lentil breeders [25]. When seed samples collected from experiments 1–3 and 7 were tested for BYMV seed-to-seedling transmission, only low rates of seed transmission were detected. These occurred in three pulse species: faba bean (0.5%), grass pea (0.2%) and dwarf chickling (0.1%) [25]

In glasshouse tests in WA in 1997–1999, when six BYMV isolates causing either necrotic or non-necrotic symptoms in narrow-leafed lupin plants (three of each type) were inoculated to 23 different genotypes belonging to faba bean (5), field pea (5), lentil (2), chickpea (3), grass pea (1), dwarf chickling (2), narbon bean (2), common vetch (2) and *Vicia bengalensis* (1), except in chickpea, none of them caused a necrotic phenotype involving shoot death like the systemic necrosis elicited by the necrotic strain in narrow-leafed lupin (Section 5.1 above). However, there were clear differences in symptom type and/or severity depending upon the BYMV isolate used and crop cultivar/species inoculated (Figure 12D–H) [47]. In faba bean, although all five cultivars were easily infected with the necrotic strain isolates LP and SV, inducing severe symptoms (Figure 12D), they were difficult to infect when inoculated with three non-necrotic isolates and necrotic isolate MI. Amongst these five cultivars, cv. Fiesta became infected most readily and developed the most severe symptoms, whereas cv. Icarus was the most difficult to infect, especially with isolate MI, which induced necrotic local lesions consistent with the presence of strain-specific localized hypersensitive resistance (Figure 12E). LP and SV belong to BYMV strain group 1, MI to strain group 2 and the non-necrotic isolates to strain group 4 (see Section 5.4). As neither LP nor SV elicited localized hypersensitive resistance in cv. Icarus but both elicited systemic hypersensitive resistance with the two resistance specificities found in narrow-leafed lupin, as with other lupin species, none of these strain groups were evident when BYMV isolates were inoculated to different faba bean genotypes. Lentil, cv. Digger became infected readily by three BYMV isolates, causing symptoms that varied from severe with necrotic strain isolate MI to mild with non-necrotic strain isolate LWh-NN. However, when plants of lentil breeding line ILL7136 were inoculated with six BYMV isolates (10 plants/isolate), four failed to infect any plants (0/10), whereas the other two isolates (LEsp-NN and LWh-NN) each infected a single plant asymptomatically (1/10). In addition, when necrotic strain isolate MI was inoculated to cv. Digger and ILL 7136 seedlings, all of the former but none of the latter became infected (Figure 12F). However, due to the two single asymptomatic infections mentioned above, the likely extreme BYMV resistance of ILL 7136 reported in the previous paragraph is not completely confirmed. Nevertheless, it can still be recommended for use by lentil breeders seeking to breed for BYMV resistance. Chickpea proved very difficult to infect, with most BYMV isolate/chickpea genotype combinations, 0/10 plants becoming infected. The exceptions were cv. Heera which became infected by MI (1/10) (Figure 12G), LEsp-NN (3/10) and LKtg-NN (4/10), cv. Sona by LP (1/10), SV (1/10) and LKtg-NN (2/10) and cv. Tyson by LEsp-NN (1/10) and LWh-NN (1/10). Symptoms in the infected plants varied from tip necrosis (Figure 12G) followed by individual shoot death to leaf pallor and mild plant stunting. In field pea, although the five cultivars inoculated were infected readily by all isolates, the symptomatic response they elicited varied from severe to symptomless. Amongst the minor pulses belonging to the genus *Vicia*, narbon bean, common vetch and purple vetch (*V. benghalensis*) were normally infected easily, with symptoms varying from severe, e.g., with isolate LP in narbon bean (Figure 12H), to mild, e.g., with MI in purple vetch. With minor pulses belonging to the genus *Lathyrus*, dwarf chickling was easily infected, with cv. Chalus developing severe symptoms (Figure 12I). However, the symptoms that appeared in grass pea were milder and this species proved much less easy to infect, with none resulting in infection by the isolates LEsp-NN and SV and the other four BYMV isolates only infecting 1/10 plants each [47]. In 2007, a very high natural BYMV incidence occurred in field pea breeding plots in northern NSW [121]. There was complete BYMV resistance (0% infection) in cvs Bundi, Cressy Blue, Glenroy, Mukta and Santi and breeding lines G-1000 and OZPB819 and partial resistance in cvs Soupa Moonlight and Yarrum (3–7% infection), but 57–100% infection in the 16 other cultivars. In follow-up glasshouse tests involving sap inoculation with faba bean BYMV isolate S, the extreme BYMV resistance in cvs Bundi, Cressy Blue, Glenroy, Moonlight, Mukta and Santi and breeding lines G-1000 and OZP819 was confirmed as none became infected. Also, Yarrum was again partially resistant. Since breeding line G-1000 carries extreme resistance to PSbMV and BYMV in field pea, it is suitable for use by field pea breeders as a virus-resistant parent [121]. Similarly, some of the BYMV-resistant lines found by McKirdy et al. [25], such as lentil ILL7163, are also suitable for use as parents in virus-resistance breeding (see previous paragraph). In 2010 and 2011, BYMV lentil screening studies in Syria (in which Australian researcher J. van Leur participated) found another lentil breeding line (ILL1949) that proved highly resistant to this virus and another three (ILL518, ILL1935 and ILL7470) were rated as resistant [122]. Makkouk et al. [108] list one resistance gene (*mo*) to BYMV in field pea and two to BYMV in faba bean (*bym-1* and *bym*-2). Also, gene *mo* is considered the same as, or linked to, PSbMV resistance gene *sbm2,* which provides strain-specific resistance to PSbMV pathotypes P2 and P3. Thus, several BYMV resistance sources and genes suitable for breeding new BYMV-resistant cultivars of field pea, lentil and faba bean are available for use in Australia.

### 6.5. Integrated Disease Management

Unlike, the situation with BYMV in narrow-leafed lupin (Section 5.7 above), there has been insufficient Australian research on control measures to devise a comprehensive IDM for any other individual BYMV/pulse pathosystem. The limited information available is described above in Section 6.3 and Section 6.4. In brief, when field experimentation with BYMV in faba bean examined the effectiveness of chemical control (from applying insecticides to the soil or foliage to kill aphid vectors) or cultural control (from non-host aphid ‘stylet cleansing’ barriers), only the latter diminished BYMV spread [17,120]. When different genotypes of cool-season pulses other than lupin were screened for BYMV resistance in the field, amongst the major pulses, field pea, faba bean and chickpea were ranked as resistant, but most genotypes of lentil were susceptible and sensitive (=vulnerable). Lentil ILL7163 possessed extreme BYMV resistance, likely to be of use to lentil breeders [25]. Also, when field pea breeding plots became naturally BYMV-infected, several cultivars and breeding lines proved resistant to this virus. Subsequent glasshouse tests confirmed this. Amongst these, breeding line G-1000 carried extreme resistance not only to BYMV, but also to PSbMV so was suitable for use as a virus-resistant parent by field pea breeders [121]. In practice, farmers cannot wait for breeding programs to deliver virus-resistant cultivars before taking action to control economically threatening virus diseases. Likewise, field experimentation on individual control measure effectiveness is time-consuming and expensive. An “interim IDM strategy”, also known as a “best-bet IDM strategy”, is required for such circumstances. Devising this involves using whatever epidemiological knowledge is accessible along with generic information on control measures known to work well with other similar combinations of virus and crop [96,97,108]. For example, the comprehensive “interim IDM strategy” devised to control seed and aphid-borne BYMV, PSbMV, AMV and CMV in lentil, chickpea, faba bean and field pea plots during breeding, selection and seeding increases sites [123]. An “interim IDM strategy” which farmers can afford and employs some of these control measures was devised to be used in WA against the same four seed-borne viruses in commercial crops of these pulses (R. A. C. Jones, unpublished). In addition, a similar approach was used to develop a generic IDM strategy against viruses that afflict pulse crops in NSW [116].

### 6.6. Main Research Achievements

Australian research on BYMV/cool-season pulse pathosystems other than the BYMV/lupin pathosystem has demonstrated the following:

- The considerable diversity of foliage symptom types ranging from mild to very severe that BYMV infection causes in different cool-season pulse species other than lupin in Australia. Seed quality defects sometimes also develop, mostly in faba bean.
- BYMV occurrence in commercial crops and experimental or breeding plots of faba bean, field pea and lentil varied widely between different years in different Australian states and regions. Faba bean was infected most often. Chickpea was rarely infected. BYMV was seed-borne in seed stocks of faba bean, field pea and lentil. In northern NSW, a very high natural BYMV incidence was recorded in field pea breeding plots in 2007 and in commercial faba bean crops in 2020. In WA, BYMV infection was also found infecting experimental or breeding plots of the minor pulses narbon bean, grass pea, dwarf chickling, bitter vetch and *L. clymenum* and a dwarf chickling crop.
- Seed yield losses caused by early BYMV infection were up to 70% with faba bean and 100% in narbon bean and dwarf chickling. ‘Very poor’ seed production was recorded in lentil and *L. ochrus*.
- Clumping of newly BYMV-infected plants in faba bean plots occurred firstly around original virus source plants, but later around secondary infection foci that developed as migrant aphid vectors spread BYMV further.
- Non-host borders (e.g., of cereals) around faba bean stands suppressed BYMV spread by acting as ‘virus cleansing’ barriers, but applying insecticides to control BYMV spread was ineffective.
- All genotypes of narbon bean, dwarf chickling, *L. ochrus* and, most of lentil, were ranked as both susceptible and sensitive to infection in field screening experiments with BYMV isolate MI in WA. However, lentil ILL7163 was ranked as highly resistant and subsequent glasshouse tests found it had extreme BYMV resistance suitable for use by Australian lentil breeders. Field pea, faba bean, common vetch and grass pea genotypes were all ranked as having resistance, whereas six out of eight chickpea genotypes were in the highly resistant category (0% infection). The two chickpea genotypes ranked as resistant were always highly sensitive, but the field pea, faba bean, chickpea, common vetch and grass pea sensitivity rankings were all genotype-dependent.
- Field and glasshouse studies with field pea in NSW reported extreme resistance to BYMV in cvs Bundi, Cressy Blue, Glenroy, Moonlight, Mukta and Santi and breeding lines G-1000 and OZP819. As G-1000 also carries extreme resistance to PSbMV, it was the most suitable for use in PSbMV-resistance breeding.

### 6.7. Further Research

Amongst the four major seed-borne viruses of cool-season pulse crops other than lupin (chickpea, faba bean, field pea and lentil), over the last three decades, surveys of commercial crops growing in different Australian states have found faba bean to be the most commonly BYMV-infected crop, sometimes developing major BYMV epidemics and yield losses. BYMV infection of field pea occurred less frequently and incidences in infected crops were mostly smaller. In contrast, although genotype screening evaluations found that lentil was by far the most BYMV-susceptible and -sensitive of these four crops, widespread BYMV infection seems lacking in commercial lentil crops. Also, although chickpea proved very sensitive to BYMV infection where infection occurred in genotype screening evaluations, it was insusceptible to this virus (only 0- < 1% infection incidences) and no commercial crop BYMV infection has been reported so far. BYMV seed-to-seedling transmission occurs in commercial Australian seed stocks of faba bean and field pea and it was also found in seed samples from experimental lentil plots. Moreover, substantial seed yield losses are reported when BYMV infects faba bean, field pea and lentil. Therefore, the main focus of future BYMV research amongst these three pulse species should be on faba bean, especially in NSW, whilst a more modest focus seems appropriate on BYMV in field pea and lentil.

With faba bean, the continued breeding of new cultivars with BYMV resistance should be prioritized (Section 6.4 above) and further epidemiological studies and field experimentation on cultural control measures resembling some of those described above with narrow-leafed lupin (Section 4, Section 5.3, Section 5.5 and Section 5.6) should be undertaken. In addition, seed-borne infection with BYMV in faba bean seed crops warrants greater attention. Currently in Australia, the only quantified rate of seed-to-seedling transmission reported for a commercial seed stock is low (0.4%) (Section 6.1), but studies in other countries reported higher BYMV seed-to-seedling transmission rates, e.g., up to 2.4% in Iran [106] and up to 9% in Japan [124]. The actual rate of BYMV seed-to-seedling transmission in seeds from infected plants (i.e., intrinsic seed transmission rate) varies depending upon the faba bean cultivar and BYMV virus isolate present [124]. Moreover, in a young faba bean crop with 2.5% seed-borne seedling infection prior to aphid vector arrival, these BYMV-infected seedlings distributed randomly throughout the crop subsequently (i.e., after aphid vectors arrived) acted as the primary inoculum source for its spread to healthy plants, resulting in a high final BYMV infection incidence [124]. Since the individual field size in which faba bean crops grow is mostly very large in Australia, instead of spread from external infection reservoirs, such seedling plants acting as internal infection foci are likely to be the principal primary BYMV source for its spread by aphids within the field. Previous assumptions that the widespread infection of faba bean crops originated from virus-infected legume pasture plants or weed hosts in neighboring fields [116] seem unlikely to be correct for a non-persistently aphid-borne virus like BYMV. This is because with such viruses, there is always a rapid decline in numbers of plants infected with increasing distance from the crop margin due to the ‘stylet cleansing’ effect when an incoming viruliferous aphid vector lands on a healthy plant (Section 5.3 and Section 5.6 above) [90,125,126,127,128]. However, when not removed by herbicide, virus-infected legume pasture and weed hosts geminating from BYMV-infected seeds in the seed bank within the same field might also provide a significant alternative internal primary BYMV infection source for spread to healthy faba bean plants. Further research is needed to establish ‘threshold’ values (see Section 5.9 above) for percentage BYMV infection in faba bean seed stocks and the extent to which seedling legume pasture or weed plants growing from seed banks within sown fields (Section 4 above) are acting as internal BYMV sources for its spread to faba bean crops. Also, Australian faba bean breeding programs should ensure they never spread BYMV by releasing seed stocks of new cultivars that are already infected.

With field pea and lentil, the current focus of regular monitoring of commercial field crops for virus infection, including for BYMV, should continue to safeguard the Australia grain legume industry against unwanted surprizes and this should include establishing the extent of high-value seed crop infection with BYMV. Australian lentil and field pea breeding programs should endeavor to include BYMV-resistant breeding lines or accessions, such the lentils ILL7163 and ILL1949 and field pea G-1000, along with lines carrying BYMV-resistance gene *mo*, as parents, so that useful BYMV virus resistance is included in newly bred cultivars intended for BYMV-prone regions. These breeding programs should also ensure that they never spread BYMV by releasing already infected seed stocks of new lentil or field pea cultivars. Epidemiological studies and field experimentation on cultural control measures with BYMV in lentil and field pea are of lower priority, so could await the outcome of such studies with faba bean. The same applies to research to establish ‘threshold’ values for percentage BYMV infection in lentil and field pea seed stocks and the extent to which seedling legume pasture or weed plants growing from seed banks within sown fields are acting as internal ‘within crop’ BYMV infection foci. Another significant arena suitable for more lentil virus research in due course is the effect of mixed virus infections involving BYMV and one or more of the other viruses commonly found infecting this crop (e.g., AMV and/or CMV).

## 7. Conclusions

This historical review provides detailed information on past Australian studies with BYMV, one of the most important seed-borne viruses of the cool-season pulse crops, lupin, field pea, faba bean and lentil. It starts by providing brief background information concerning this virus in Australia, including when it was first reported in 1943, its broad natural host range across dicotyledonous and monocotyledonous plants, different aphid vector species, its presence in different states and territories and the diversity not only in the symptoms it causes in different host species, but also between its biologically distinct strains. An account is then provided of past studies on nt sequences from Australian BYMV isolates and what phylogenetic analysis has revealed about their diversity. This is followed by information about the wide range of alternative hosts from which BYMV can spread to cool-season pulse crops. These host species include sown and naturalized pasture legumes, perennial forage legumes, native perennial legumes and orchids, native species belonging to several other families and ornamental plants. Field experiments established the susceptibilities and sensitivities of many sown and naturalized annual pasture legume species to BYMV infection and the occurrence of seed-to-seedling transmission in them. Phytosanitary measures involving the removal of infected external and internal alternative pastures and weed hosts before sowing fields with cool-season pulse crops and on avoiding sowing infected seed stocks of pasture species in nearby fields, therefore, need to be included within IDM tactics against BYMV.

The lupin/BYMV pathosystem has been investigated to a much greater extent than any other cool-season pulse/BYMV pathosystem combination in Australia. What the many past studies found on this subject is covered comprehensively in this review. After a brief introduction describing the occurrence of BYMV in lupin in different Australian states and its symptomatology in different lupin species, this component of the text is subdivided into nine sub-sections entitled: ‘necrotic and non-necrotic strains’, ‘BPS’, ‘cultural control’, ‘host resistance’, ‘temporal and spatial dynamics of spread’, ‘epidemic drivers and forecasting’, ‘integrated disease management’, ‘main research achievements’ and ‘further research’. Except in the ‘host resistance’ subsection, most of the research concerns BYMV in narrow-leafed lupin. This is because this is the most economically important lupin crop species grown in Australia and is, therefore, the most studied. The field experiments on ‘cultural control’, ‘host resistance’ and the ‘temporal and spatial dynamics of BYMV spread’ provided profound insights into the BYMV narrow-leafed lupin disease combination which enabled its epidemic drivers to be well understood. This in turn made it possible to devise a forecasting model and comprehensive IDM strategies and provide focused recommendations for future research into further improvements regarding its management.

Information concerning what is known about the less thoroughly investigated cool-season pulse/BYMV pathosystems in Australia, especially in the three main crops threatened, faba bean, field pea and lentil, is then provided. After a brief introduction describing BYMV’s symptomatology in these three pulses, chickpea and minor cool-season pulses, this text component is subdivided into seven different sub-sections entitled: ‘occurrence in plots, crops and seed stocks’, ‘seed yield losses and patterns of spread’, ‘chemical and cultural control’, ‘host resistance’, ‘integrated disease management’, ‘main research achievements’ and ‘further research’. The ‘host resistance’ section provides important findings relevant to breeding BYMV-resistant cultivars of faba bean, field pea and lentil suited to Australian conditions. Currently, however, with these cool-season pulse pathosystems, there is insufficient information and understanding of their epidemic drivers to enable the development of comprehensive and effective forecasting models and IDM strategies to optimize their control. Providing such information in the future for faba bean, field pea and lentil would require the allocation of research resources resembling those provided in the past to investigate the BYMV/narrow-leafed lupin pathosystem.

Section 5.8 and Section 6.6 provide summaries of key Australian research findings with each of each of the lupin crop species/BYMV pathosystems and other cool-season pulse/BYMV pathosystems, respectively. Perhaps the most noteworthy finding arising from the field and glasshouse studies is that strain-specific systemic hypersensitive resistance operates at the plant population level, limiting further virus spread by killing infected plants, thereby removing them as internal virus infection sources for further virus spread by vectors which, in turn, diminishes the overall yield losses (Section 5.1, Section 5.4 and Section 5.5). This result with a BYMV/legume pathosystem has wide implications for all other plant virus/host species pathosysytems in which systemic hypersensitive resistance is operating in the field. Section 5.9 and Section 6.7 provide future research recommendations for each of the lupin crop species/BYMV pathosystems and other cool-season pulse/BYMV pathosystems, respectively. Together, Section 5.8, Section 5.9, Section 6.6 and Section 6.7 will help the reader understand exactly what has been achieved and what research is still required. The extensive collection of images taken of past field experimentation will help develop an understanding of the kinds of approaches that will be required in the future.

Generic recommendations for future seed-borne virus disease research in Australia were discussed in Volume 1. These included addressing concerns relating to increasing biosecurity incursions and difficulties in managing virus disease epidemics arising from climate instability and alterations in agricultural practices. They also included adoption of exciting new technologies such as: (i) enhancing precision agriculture by employing remote sensing and machine learning to help identify virus-diseased plants, establish vector and virus disease levels and precisely target localized virus infection and vector foci within crops with chemical control measures and (ii) integrating speed breeding with genetic modification by RNAi and CRISPR to improve virus resistance in new cultivars.

## Data Availability

Not applicable for reviews based entirely upon previously published information.
